# Evaluating the role of 2-hydroxyestradiol in modulating TNF-α signaling and its implications in rheumatoid arthritis

**DOI:** 10.1042/CS20241917

**Published:** 2025-09-22

**Authors:** Prachi Agnihotri, Mohd Saquib, Ajit Kumar, Lovely Joshi, Debolina Chakraborty, Ashish Sarkar, Vijay Kumar, Sagarika Biswas

**Affiliations:** 1 Council of Scientific & Industrial Research (CSIR)-Institute of Genomics and Integrative Biology, Mall Road, Delhi University Campus, Delhi, 110007, India; 2 Academy of Scientific and Innovative Research (AcSIR), Ghaziabad, 201002, India; 3 All India Institute of Medical Sciences, Ansari Nagar, New Delhi, 110029, India

**Keywords:** 2-hydroxyestradiol, estrogen metabolites, inflammatory pathways, rheumatoid arthritis, TNF-α

## Abstract

Estrogen metabolites, a subset of female sex hormones, are known to play a role in autoimmunity and the inflammatory response associated with rheumatoid arthritis (RA). Current therapeutic approaches have focused on the development of small molecules that can easily alter intracellular components within a signaling pathway, thereby selectively targeting the disease physiology. In the present study, small molecule 2-hydroxyestradiol (2-OHE2) was utilized to investigate the potential anti-inflammatory effects by *in vitro* studies using primary fibroblast-like synoviocytes (RA-FLS) and *in vivo* studies using collagen-induced arthritis (CIA) rat model. A biophysical interaction study between 2-OHE2 and TNF-α was conducted using tyrosine quenching and thermal shift assay, and the maximum fluorescence quenching of the tyrosine residue was observed at a ligand concentration of 1 mM. Further, the impact of 2-OHE2 on the TNF-α signaling pathway was evaluated in RA-FLS, focusing on the inhibition of inflammation, cell proliferation, and apoptosis activation, using Western blotting, quantitative real-time polymerase chain reaction (qRT-PCR), caspase 3/7 activity, scratch assays, and immunocytochemistry. We found that 2-OHE2 effectively down-regulates inflammatory mediators, adhesion markers, and metalloproteinases and regulates apoptotic markers in RA-FLS. 2-OHE2 also exhibited anti-inflammatory effects in the CIA rat model. Our findings indicate that 2-OHE2 may be a promising potential anti-inflammatory compound. It targets TNF-α signaling, exhibiting pro-apoptotic and anti-proliferative effects, suggesting that 2-OHE2 could have therapeutic implications in the pathophysiology of RA.

## Introduction

Rheumatoid arthritis (RA) is an autoimmune disease affecting nearly 1% of the global population [1]. The available diagnostic markers, namely ACPA and RF factor, are non-specific with limited sensitivity and specificity [2]. The recommended treatment for this disease is generally disease-modifying antirheumatic drugs (DMARDs) and non-steroidal anti-inflammatory drugs (NSAIDs). Currently, biologics have also been developed as injectable medications targeting TNF-α. The pathogenesis of RA involves several proinflammatory cytokines, with TNF-α being the most notable. However, the prolonged usage of biologics has been linked to a range of side effects [3]. Hence, there is a need to explore more effective alternative approaches that entail fewer side effects.

Targeting TNF-α and its binding to TNFR has therapeutic potential due to well-established mechanism that activates NF-kB-p50/65 proteins, producing pro-inflammatory cytokines and proteins in RA [4]. Studies have found selective small-molecule antagonists of TNF-α that exhibit inhibitory effects in RA [5]. Metabolites are small molecules, playing roles in metabolic reactions, signaling, and energy production that are formed as byproducts of metabolic pathways. They serve as checkpoints for cellular changes and provide insight into disease initiation [6]. Recently, various metabolites have been investigated, which are reported to hold therapeutic potential and important regulatory mechanisms for various diseases [7]. In the recent past, we uncovered one such estrogen metabolite, 2-hydroxyestradiol (2-OHE2), holding promising binding ability with TNF-α and proposed its efficiency as a selective small-molecule antagonist of TNF-α [8,9].

Estrogen and progesterone are key to female physiology, shaping physical traits, and managing reproduction. Alongside corticosteroids and androgens, these sex steroids perform specific functions in the body [10]. Estrogen deficiency can significantly affect fertility, contributing to reproductive challenges [11], and it plays a key role in inflammatory diseases, including RA, with females exhibiting a higher incidence rate than males (3:1). Studies have shown that estrogen deficiency leads to joint inflammation and bone erosion [12,13]. It is crucial to understand estrogen metabolism within the body. This complex process takes place in the liver, producing metabolites that play a vital role in energy conversion, signaling, and epigenetic effects. The resulting metabolites of estrogen are known as catechol estrogens (CEs), which are formed through the hydroxylation of estrogen [14]. CEs are differentially regulated in RA, which presents promising opportunities for research and advancement [15]. Understanding estrogen metabolites and investigating their role in RA pathophysiology is vital, as it may reveal their potential to revolutionize health. Certain metabolites, such as itaconate and adenosine, have been studied for their anti-inflammatory properties in ocular bacterial infections. They help to limit the harm caused by inflammation, oxidants, and proteases in a specific area [16]. Moreover, itaconate has been observed to regulate HIF1 production and succinate levels in activated macrophages and to reduce the secretion of proinflammatory cytokines [17]. 2-OHE2 interacts with TNF-α, suggesting that 2-OHE2 may regulate the inflammatory responses initiated by TNF-α signaling, highlighting its potential therapeutic implications in the pathogenesis of RA. In this study, the metabolite 2-OHE2 was investigated through biophysical interactions, followed by *in vitro* and *in vivo* experiments, validating its role in monitoring cellular inflammatory mechanisms in managing the pathobiology of RA.

## Materials and methods

### Plasmid construction and protein purification

Plasmid constructed, encoding 77–233 amino acid region of human TNF-α, was generated by cloning codon-optimized full-length human TNF-α gblocks (IDT) into the pET-28a vector. Confirmation of successful cloning was performed through Sanger sequencing. Subsequently, overexpression was conducted in *E. coli* (Rosetta) cells, induced at OD 600 nM~0.6–0.7 with 0.2 mM IPTG, and incubated at 20°C for 22–24 h [18]. Following cell harvesting, the cells were resuspended in lysis buffer (50 mM phosphate buffer, 150 mM NaCl, 10 mM imidazole) and lysed via sonication. The resulting clear supernatant was subjected to Ni_2_SO_4_-charged HiTrap columns (GE Healthcare) and washed with a buffer (50 mM phosphate buffer, 150 mM NaCl, and 20 mM imidazole). Subsequently, the protein was eluted using a buffer comprising 50 mM phosphate buffer, 150 mM NaCl, and 250 mM imidazole. Purification of protein was achieved through size exclusion chromatography (FPLC) using Superdex 75 pg (Cytiva) in a binding buffer (137 mM NaCl, 8 mM Na_2_HPO_4_ (pH 7.4), 2.7 mM KCl, 1.5 mM KH_2_PO_4_, 1 mM EDTA, and 1 mM TCEP). Homogeneity of the purified protein was confirmed using 12% SDS-PAGE, and the final product was stored at 4°C until further use.

### Tyrosine fluorescence quenching

Fluorescence spectroscopic experiments were performed on Fluorolog-3 Spectrophotometer LS55 (Horiba Jobin Yvon Spex ®) equipped with a xenon lamp source. Spectra were recorded at 25℃. An excitation wavelength (λex) of 275 nm was used for tyrosine quenching, and emission spectra were recorded for the wavelength range from 290 nm to 400 nm. The free TNF-α (10 μM) and 2-OHE2 at increasing ligand concentrations (ranging from 25 μM to 1 mM) were prepared in a binding buffer and incubated for 10 min before recording their fluorescence spectra. Fluorescence spectra were acquired for free protein and protein–ligand complexes at increasing ligand concentrations in quartz cuvette. Excitation and emission slit widths were maintained at 5 nm. The binding of 2-OHE2 to TNF-α was characterized by monitoring the quenching of tyrosine fluorescence upon increasing concentrations of the ligand to TNF-α [19].

### Thermal shift assay (SYPRO Orange)

Fluorescence-probed thermal denaturation assay was performed to evaluate the compounds’ effect on protein’s thermal stability as a measure of compound binding. The reaction mixture contained 3  µl 30XSYPRO^®^ Orange dye (Invitrogen), 7  µl TNF-α (1.5 μM), 18.5  μl PBS (pH 7.4), and 1.5  μl compound (2-OHE2 at 2  mM in DMSO). The mixture (10  µl) was dispensed in triplicate into a 96 polymerase chain reaction (PCR) optical well plate and was run on a Life Cycler 480 II Real PCR System (Roche Technologies). The PCR system heating device was set at 20–99°C with a ramp rate of 1.1°C min^−1^. The increase in the fluorescence intensity was plotted as a function of temperature, and the *T*_m_ value was calculated as the midpoint of this denatured curve (determined as the point of inflection) [20].

### *In vitro* analysis

#### Sample collection

Biopsy tissue samples were collected from RA (*n* = 8) from the Orthopedic Department, All India Institute of Medical Sciences (AIIMS), New Delhi, India, who met the revised 2010 American College of Rheumatology (ACR) and European League Against Rheumatism (EULAR) Rheumatism diagnosis criteria [21]. Pregnant women, alcoholic patients, and patients with other diseases such as diabetes, cardiovascular disease, or any other inflammatory diseases were excluded. Biopsy synovium samples were collected from patients who had undergone surgery as a treatment option. The medical history of each patient was collected (Supplementary Table S1).

#### Isolation and culture of RA fibroblast-like synoviocytes (FLS)

After collection of biopsy synovium, tissues were washed with PBS twice, and synovium samples (≈20 mg) were separated from the adipose tissue, chopped finely, treated with collagenase (0.5 mg/gm of tissue), dissolved in 30 ml complete Dulbecco’s Modified Eagle Medium (DMEM), and incubated for 12–18 h. The tissue suspension was then passed through the cell strainer (100 µm pore size, BD) and cultured and maintained for two passages in a T-75 tissue culture flask in complete DMEM; thereafter, the cells were used for experiments (third to fifth passage) [22]. RA fibroblast-like synoviocytes (RA-FLS) were cultured in DMEM supplemented with 10% Fetal Bovine Serum (FBS) at 37°C under a humidified atmosphere containing 5% CO_2_ [23].

#### Cell survivability assay

RA-FLS were seeded in 96-well culture plates. After 70–80% confluence, cells were treated with serially diluted 2-OHE2 (100–0.312 μM range) in serum-free media for 24 h. The cell viability was measured using 3-(4, 5-dimethylthiazol-2-yl)-2, 5-diphenyl tetrazolium bromide (MTT) assay, as per the manufacturer’s instructions, and absorbance was measured at 540 nm in a spectrophotometer (Molecular Devices). Dimethyl sulfoxide (DMSO) (20%) was used as a toxic control [24].

#### RT-PCR assay

RA-FLS cells were cultured in a T–25 culture flask. After reaching 70–80% confluency, cells were induced with 1.25 μM 2-OHE2 in serum-free media for 24 h. Total RNA was isolated using Tri-Xtract reagent (G Biosciences) according to the manufacturer’s instructions and 1 µg of total RNA was used for cDNA preparation using cDNA Synthesis Kit (G Biosciences). The transcribed cDNAs were mixed with HOT FIREPol EvaGreen qPCR Master mix (SOLIS DYNE), and the level of mRNA expression was evaluated using an RT-PCR (Roche light cycler 480 II detection system) using human-specific primer sequences (Supplementary Table S2). The data were normalized with GAPDH used as an internal control and analyzed quantitatively using the 2-^ΔΔCT^ formula [24].

#### Total protein extraction and Western blotting

RA-FLS cells were cultured until they reached 70–80% confluency. Cells were treated with 2-OHE2 for 24 h in serum-free media. The cell lysate was extracted using ice-cold radioimmunoprecipitation assay (RIPA) cell lysis buffer consisting of protease and phosphatase inhibitors. The protein (20 μg) concentration was estimated by Bradford protein assay, separated on 12% SDS-PAGE, and electro-transferred using a Trans-Blot Semi-dry transfer unit (Bio-Rad, U.S.A.). The membranes were blocked for 4 h at room temperature (RT) with 5% BSA (Sigma-Aldrich, U.S.A.) followed by overnight (O/N) incubation individually with diluted (1:4000) primary antibodies (p65, VEGF, Caspase3, Bax, Bcl2, Cytc, MMP9, IκBα, Ki67 (Santa Cruz), CDH11, VCAM-1 (G-Bioscience) and N-CAD (cloud clone) p-p65 (CST), FGF (Abcam) and loading control GAPDH , β-actin and Vinculin (Santa Cruz) at 4°C. The blots were then washed and incubated for 1 h with horseradish peroxidase (HRP) conjugated anti-mouse and anti-rabbit as secondary antibody (Jackson, U.S.A.), (1:8000), developed using enhanced chemiluminescence (ECL) (Thermo Scientific, Pierce, U.S.A.), and scanned using ChemiDocTM MP Imaging system (Bio-Rad, U.S.A.) [25].

#### Caspase-Glo 3/7 activity assay

RA-FLS cells were treated with 2-OHE2 for 24 h in serum-free media subjected to Caspase 3/7 activities measurement with Caspase-Glo assay kit (Promega, Madison U.S.A.). The plates were allowed to equilibrate at RT for 30 min. Caspase-Glo reagent (100 μl) was then added to each well, gently mixed the content using a plate shaker at 300–500 rpm for 30 s, and incubated in a CO_2_ incubator for 2 h. The luminescence of each sample was measured in a plate-reading luminometer (Tecan-icontrol) with parameters of 1 min lag time and 0.5 s/well read time. The experiments were performed in triplicate and repeated on two separately initiated cultures [26,27].

#### Scratch assay analysis

The cells were grown in six-well tissue culture plates up to 80% confluency. A vertical scratch was drawn with a pipette tip at the center of the flask, and each scratch area was measured before and after treatments. The cells were then treated with 2-OHE2 (1.25 μM). The cells were further grown in media for another 48 h, followed by the acquisition of bright-field images using a Nikon Eclipse 650 (NIKON, Tokyo, Japan) at ×10 magnification. The images were then analyzed using ImageJ software, and the scar area was measured by selecting the freehand tool application in ImageJ software [28].

#### Immunocytochemistry

Both treated and untreated (Control) RA-FLS cells were fixed on a glass slide with 4% paraformaldehyde solution for 15 mins, followed by quenching using quenching buffer (1% glycine in 1X PBS) for 5 min and permeabilization using 0.1% Triton X-100/PBS for 15 min. Cells were blocked for O/N with 3% BSA; slides were stained with Abs to TNF-α (1:100; Santa Cruz), p65 (1:100; Santa Cruz), and TNF-R1 (1:300; CST) for 2.5 h at RT. Each slide was washed three times with PBST and incubated with a Donkey anti-rabbit IgG(H + L) Alexa Fluor 647 Ab (1:600; Invitrogen) and Goat anti-mouse IgG(H + L) Alexa Fluor 488 Ab (1:600; Invitrogen) for 1.30 h at RT for tagging with the two proteins respectively. 4′,6-diamidino-2-phenylindole (DAPI) was used to stain the nuclei, and the glass slides were mounted with Vectashield Antifade Mounting Medium (Vector Laboratories). A confocal microscope was used to examine the stained cells (LEICA DMi8; TCS SP8, Germany). The raw images were analyzed by LAS X microscope software and quantitatively using Image J software [9].

### *In vivo* studies

#### Development of collagen-induced arthritis (CIA) rat model

Female Wistar rats of four to six weeks (50–80 g) were purchased from the National Institute of Nutrition, Hyderabad, India. The animals were maintained, and experiments were performed at the Institute’s in-house animal facility with a controlled environment (25 ± 2°C), 42% humidity, under a 12 h light–dark cycle, and were acclimatized for 1 week.

After a week of acclimatization, the animals were randomly divided into four groups (*n* = 4). The untreated group/ healthy control (HC) (Group 1), collagen-induced arthritis (CIA) (Group 2), vehicle control (VC+CIA) (Group 3), and 2-OHE2 treated (CIA + 2-OHE2) (Group 4). CIA rats (Except HC group) were then induced with 2 mg/ml collagen (Type II) from chicken (Sigma, U.S.A.) dissolved in 0.01 M acetic acid and combined (1:1) with complete adjuvant (Sigma, U.S.A.).

2-OHE2 was administered at 800 μg/kg [29] of rat body weight mixed with corn oil/ benzyl alcohol (95:5 v/v) and was injected subcutaneously. Subcutaneous administration often causes minimal pain or discomfort, and the administration of material is non-irritant [30,31]. The collagen dosage was administered through the tail vein on the 7th and 14th days, 2-OHE2 dose was administered a day before the induction of second CIA and every 3rd day until the 28th day [27]. On the 29th day, rats were sacrificed using a combination of Thiopentone and Xylazine (3:1). Blood and synovium were collected for further evaluation.

#### Measurement of CIA development in experimental groups and detection of RA

Throughout the study, the body weight and paw volume were measured, and clinical arthritis scores such as paw redness, swelling, and arthritis index (AI) were assessed in individual animals to monitor disease progression from day 0th to day 28th. The arthritis index (AI%) was measured and calculated using the below-mentioned formula [32].


$$ArthitisIndex(\%)=\frac{HindpawvolumeondayX-Hindpawvolumeonday0}{Hindpawvolumeonday0}\times 100$$

The macroscopic arthritis score was determined by macroscopic examination of swelling, edema, and redness in all four paws of CIA rats. The severity was graded on 1 to 4 scale: 1 indicates no obvious edema, swelling, or redness, 2 indicates moderately involved joints, 3 indicates highly involved joints with edema, redness, and swelling, and 4 indicates severely affected joints with edema, swelling, and redness. A plethysmometer was used to calculate and quantify the swelling of the joints. Changes in body weight were monitored and calculated by subtracting body weight of day 0 from day 28. On day 29, the spleen and liver were removed and weighed immediately, after which the splenic index and liver index were calculated for each rat as the ratio of the spleen/liver:body weight.

#### Enzyme-linked immunosorbent assay (ELISA)

Rat plasma was separated and added (100 µl) to the pre-coated ELISA plate, followed by the manufacturer’s guidelines. TNF-α, IL-1β, and IL-6 cytokines were quantified using ELISA kits (ELK Biotechnology, China) [32].

#### Hematoxylin and eosin (H&E) staining

Rat synovium was sliced and fixed in 10% formalin, fixed in the paraffin block, and sliced (5 µm thick) using a microtome. The slices were mounted on slides, deparaffinized, rehydrated with distilled water, and stained (1 min) with hematoxylin. The slides were then dehydrated with ethanol, xylene washed, decolorized with 0.05% acid alcohol, rinsed, counter-stained with alcoholic Eosin (30 s), mounted coverslips, and viewed under a Nikon microscope. Images of the slides at 10× magnification were taken, and ImageJ software was used to analyze the images [33].

#### Statistical analysis

GraphPad Prism 9.5 Software (U.S.A.) was used to calculate the significant difference and graphing. To compare data from two or more groups, the Student’s *t*-test was conducted, including Mann–Whitney *U* and analysis of variance (ANOVA), and *P*<0.05 was considered significant, with mean ± S.D., and only statistically significant (*P*≤0.05) values were considered.

## Results

### 2-OHE2 binds to a tyrosine residue in the TNF-α active site, affecting its stability

**Figure 1:**
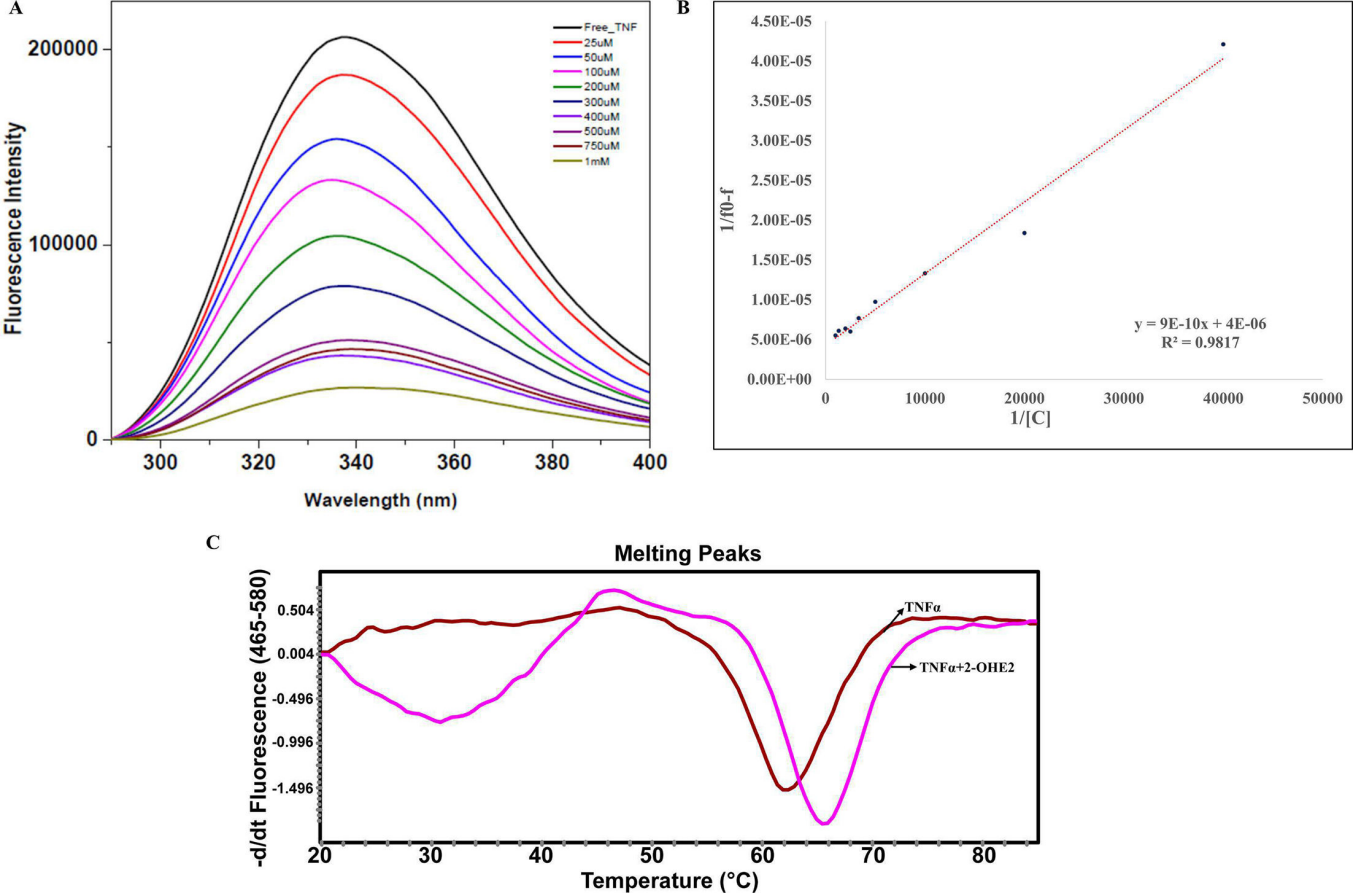
Tyrosine quenching of TNF-α interaction with 2-OHE2. (**A**) Fluorescence quenching of TNF-α with different concentrations of 2-OHE2 (25 μM to 1 mM) indicates a direct interaction with the active tyrosine residues, becomes quenched upon interaction at maximum concentration of ligand (1 mM). (**B**) Benesi–Hildebrand plot of TNF-α with varied concentrations of 2-OHE2 at 298 K. The associate constant (Ka) was calculated to be 4.44 × 10^3^ M^−1^. (**C**) Thermal unfolding of TNF-α is monitored using SYPRO Orange: Thermal denaturation of the TNF-α at pH 7.0 with 2-OHE2. First-order derivatives were generated as a function of temperature. Data were collected in the presence of TNF-α alone and with ligand concentrations, leading to a rightward shift in the unfolding transition. 2-OHE2, 2-hydroxyestradiol; TNF-α, tumor necrosis factor alpha.

It has been previously reported that Tyr residues are crucial for the functional activity of TNF-α [34,35]. Further, the active site of TNF-α also involves interactions with Leu57, Tyr59, Tyr119, and Tyr151, primarily through hydrophobic interactions, indicating that engaging with these residues might serve as precise inhibition of the target protein [36]. In accordance with this, we carried out a tyrosine quenching assay, indicating a substantial reduction in tyrosine fluorescence of TNF-α upon titrating with 2-OHE2 (Figure 1A). TNF-α protein was purified by FPLC (Supplementary Figure S1 and S2) and fractions were eluted (Supplementary Figure S3). The association constant (*Ka*) of the TNF-α-2-OHE2 system was computed from the Benesi–Hildebrand equation [37], and it was found to be *Ka* = 4.44 x 10^3^ M^−1^. (Figure 1B) inferred the stability of the complex. These findings strongly suggest that 2-OHE2 could directly bind to the active site of TNF-α, thus affecting its stability.

### 2-OHE2 effect on thermal denaturation of TNF-α

The thermofluor assay, which utilizes SYPRO Orange dye, is suitable for most soluble proteins that are well-folded and feature a relatively large hydrophobic core [38]. The thermal stability of TNF-α was therefore evaluated by thermal shift assay (TSA) with our identified ligand 2-OHE2. The raw denaturation curves of TNF-α (brown) and TNF-α with 2-OHE2 (magenta) and their first-order derivative elaborated with melting temperature (*T*_m_) were shown (Figure 1C). The fluorescence peak shifts toward higher temperatures as the ligand binds, resulting in an increase in fluorescence that can be observed as a negative derivative. The *T*_m_ of the TNF-α was determined to be 62 ± 1℃, increased to 66 ± 1℃ in the presence of 2-OHE2, as revealed from the curve (Figure 1C)

### RA-FLS cells viability analysis

**Figure 2:**
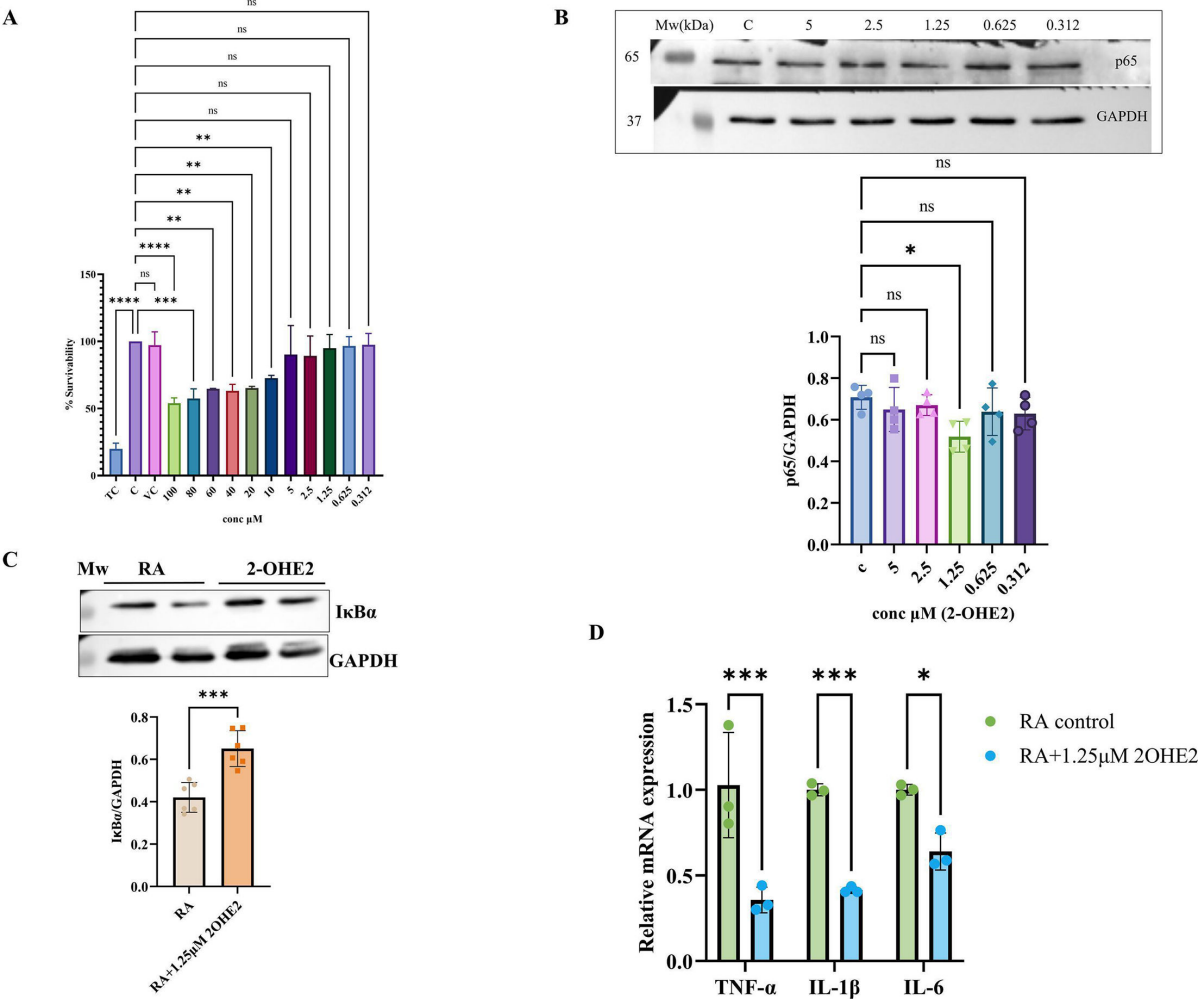
Cellular viability of 2-OHE2 and its effect on inflammation in RA-FLS. **(A**) The cell viability test was performed by the MTT assay. The bar represents the cell viability results of RA-FLS cells treated with 2-OHE2 (100–0.312 μM) for 24 h. (**B**) The expression level of p65 observed in RA-FLS by Western blot, pretreated with serial dilutions of 2-OHE2 (5–0.312 μM) for 24 h, revealed significantly (*P*=0.0199) down-regulated expression at 2-OHE2 at 1.25 μM. (**C**) The expression level of IκBα observed in RA-FLS by Western blot, pretreated with 1.25 μM 2-OHE2 for 24 h, found significantly (*P*=0.0004) up-regulated (~1.75 fold). (**D**) mRNA expression analysis of cytokines TNF-α, IL-1β, IL-6 by qRT-PCR found a significantly decreased expression in RA-FLS cells pretreated with 1.25 μM 2-OHE2 for 24 h. Values are presented as the mean ± SEM (*n* = 3). *P< 0.05, **P<0.01, ***P<0.001, and ****P<0.0001. 2-OHE2, 2-hydroxyestradiol; IκBα, nuclear factor of kappa light polypeptide gene enhancer in B-cells inhibitor α; IL-1β, interleukin 1 beta; IL-6, interleukin -6; MTT, 3-(4, 5-dimethylthiazol-2-yl)-2, 5-diphenyl tetrazolium bromide; NF-κB p65, nuclear factor kappa-light-chain-enhancer of activated B cells; qRT-PCR, quantitative real-time polymerase chain reaction; RA-FLS, rheumatoid arthritis fibroblast-like synoviocytes; TNF-α, tumor necrosis factor alpha.

We conducted MTT assay to measure the cell viability of RA-FLS, pre-treated with 2-OHE2 at concentrations ranging from 100 μM to 0.312 μM for 24 h. The bar graph represents the percentage (%) of cell survivability after the induction of cells. Our results indicated that concentrations above 5 μM were toxic to cells. Therefore, we have selected concentrations below 5 μM for further analysis (Figure 2A).

### 2-OHE2 has a potent anti-inflammatory effect in RA-FLS by inhibiting TNF-α signaling

TNF-α typically induces NF-κB signaling by translocating the p65 (RelA) DNA binding factor to the nucleus [39]. We analyzed the NFκB/(p65) expression level, which is a prominent inflammatory mediator in RA-FLS. Western analysis of RA-FLS showed a significant decrease in the expression of NFκB (p65) at a concentration of 1.25 μM (*P*=0.0199) (Figure 2B). We also analyzed the levels of p65 in the cytoplasmic and p-p65 in the nuclear fraction [40] and after stimulation with 2-OHE2 for 24 h were found down-regulated at 1.25 μM concentrations (Supplementary Figure S4A and S4B). The expression of p65 (green color) was observed to be significantly high in the merged image of the control cells compared with the merged image of 2-OHE2-treated cells at this dose (Supplementary Figure S5). Therefore, the dose of 1.25 μM of 2-OHE2 was selected for further experiments.

In general, the transcription factor NF-κB exists in a cytosolic ‘resting’ state as a dimer, which is inactivated by its association with inhibitors of κB (IκB). This prevents the dimer from binding to target sites in the nucleus and thus initiates gene transcription [41]. We found that at a concentration of 1.25 μM, 2-OHE2 significantly increased the levels of IκBα (~1.75 fold), suggesting that 2-OHE2 may inhibit the activation and nuclear translocation of NF-κB. (Figure 2C)

Reports suggested that the NF-κB pathway is important in regulating the release of proinflammatory cytokines in RA [42]. We, therefore, analyzed the mRNA expression of pro-inflammatory mediators (IL-6, IL-1β, and TNF-α) in RA-FLS cells and observed that 2-OHE2 at 1.25 μM significantly reduced the expression of pro-inflammatory mediators (**Figure 2D),** suggesting that 2-OHE2 has the potential to suppress inflammation by targeting TNF-α.

### 2-OHE2 inhibits proliferation and adhesion in RA-FLS by impeding signaling initiated by the interaction between TNF-α and TNF-R1

**Figure 3:**
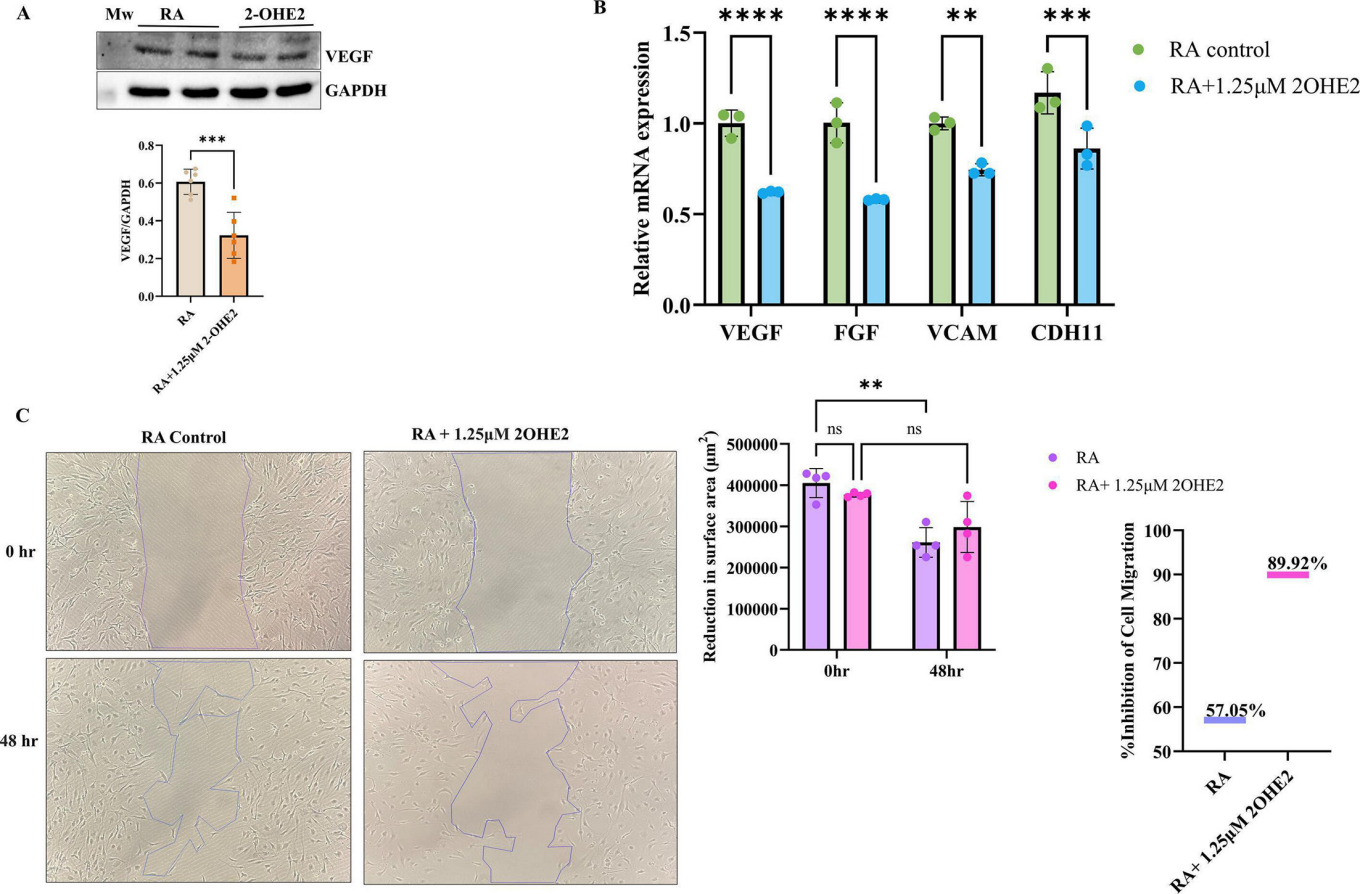
Effect of 2-OHE2 on cellular adhesion and migration in RA-FLS. **(A**) The expression level of VEGF observed in RA-FLS by Western Blot, pre-treated with 1.25 μM 2-OHE2 for 24 h, found significantly (*P*=0.0005) up-regulated (~0.5 fold). (**B**) mRNA expression analysis of adhesion factors VEGF, FGF, VCAM-1, CDH11 by qRT-PCR found a significant decrease in RA-FLS cells pretreated with 1.25 μM 2-OHE2 for 24 h. (**C) Scratch assay:** Migration ability of RA-FLS by Scratch assay after stimulation with 1.25 μM 2-OHE2 for 24 h. Images were taken at 0 h and 48 h. The results showed that the % inhibition of migration in control was 57.05%, in 2-OHE2 treated cells 89% at 48 h compared with 0 h. Statistical analysis used the Student’s *t*-test, including Mann–Whitney *U*, and analysis of variance (ANOVA), values are presented as the mean ± SEM (*n* = 3). **P<0.01, ***P<0.001 and ****P<0.0001. 2-OHE2, 2-hydroxyestradiol; CDH11, cadherin-11; FGF, fibroblast growth factors; qRT-PCR, quantitative real-time polymerase chain reaction; RA-FLS, rheumatoid arthritis fibroblast-like synoviocytes; VCAM-1, vascular cell adhesion molecule 1; VEGF, vascular endothelial growth factor.

The role of TNF-α in promoting up-regulation of adhesion molecules and activation of signaling through TNF-R1 is well-established [43]. TNF-α exerts its antiangiogenic effect by modulating the VEGF-specific angiogenic pathway [38,44]. To assess the expression of VEGF, Western blot analysis was performed, and a significant down-regulation (~0.5 fold) of its expression in RA-FLS was observed upon treatment with 2-OHE2 at 1.25 μM (Figure 3A). Further, a marked decrease in the mRNA expression of VEGF and associated adhesion markers FGF, VCAM-1, and CDH11 (Figure 3B) was also observed. We found a decreased expression of Ki67 (~0.6 fold) and N-CAD (~0.5 fold) after the induction of 2-OHE2, as these are important markers for proliferation and migration [45,46]. (Supplementary Figure S6A,B). Moreover, a decreased expression of FGF (~0.6 fold), CDH11 (~0.8 fold), and VCAM-1 (~0.75 fold) was observed following the induction of 2-OHE2, as these are essential markers for adhesion. (Supplementary Figure S7A,B and C).

Next, the wound healing assay was carried out to determine the effect of 2-OHE2 on cell migration and invasion capability. The results indicated a decrease in the migration of cells when treated with 2-OHE2 (1.25 μM) at 48 h compared with the untreated cells (RA Control), indicating that 2-OHE2 can inhibit cell migration and invasion of cells. The gaps marked in the control cells were almost filled with cell migration, while the cell migration ability in the 2-OHE2 (1.25 μM)-treated group was found to be decreased. The % inhibition of cell migration was 57% in control cells at 48 h, and the treatment with 2-OHE2 (1.25 μM) showed 89% inhibition in cell migration (Figure 3C). It determines the antiproliferative capability of 2-OHE2 as it inhibits cell migration under inflammatory conditions.

### 2-OHE2 induces cellular apoptosis

**Figure 4:**
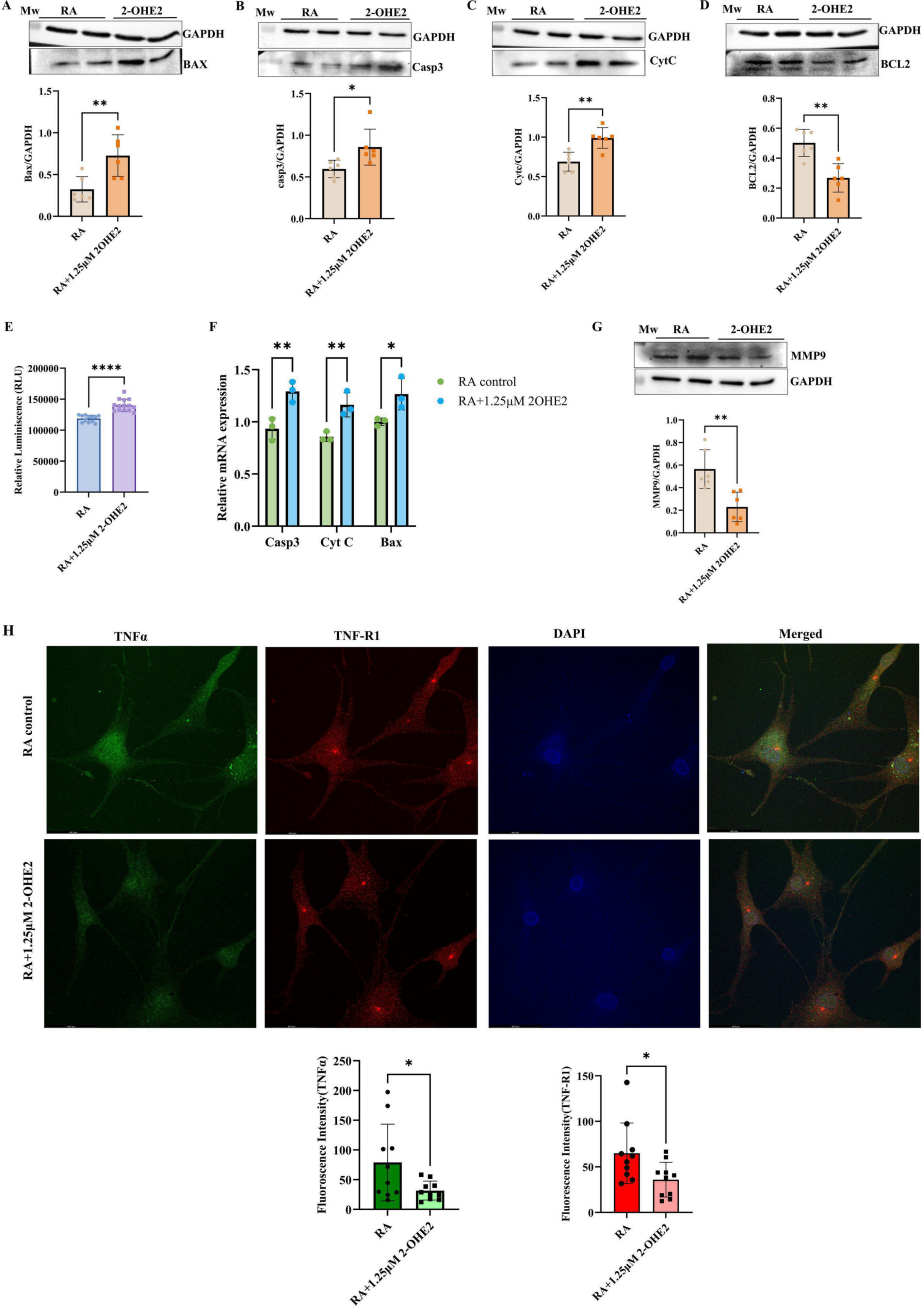
2-OHE2 exerts pro-apoptotic effects and disrupts the interaction between TNF-α and TNF-R1 in RA-FLS. The expression level of BAX, Caspase 3, Cytc, and Bcl2 was observed in RA-FLS by Western blot, pretreated with 1.25 μM 2-OHE2 for 24 h. The level of proapoptotic proteins, (**A**) BAX (*P*=0.0066) (~2.3 fold), (**B**) Caspase3 (*P*=0.0231) (~1.5 fold)**, (C**) Cytc (*P*=0.0020) (~1.3 fold)**,** was found to be significantly up-regulated (**D**) The level of anti-apoptotic proteins Bcl2 (*P*=0.0014) (~0.42 fold) was significantly down-regulated. (**E**) Increased relative luminescence ( ~ 1.16 fold) of caspase-3/7 was measured in RA-FLS cells by the Caspase-Glo ® 3/7 assay in pre-treated with 1.25 μM 2-OHE2 for 24 h. (**F**) mRNA expression analysis of proapoptotic proteins BAX, Caspase 3, Cytc by qRT-PCR found a significant up-regulation in RA-FLS cells pretreated with 1.25 μM 2-OHE2 for 24 h. (**G**) The expression level of MMP-9 observed in RA-FLS by Western blot, pretreated with 1.25 μM 2-OHE2 for 24 h, was found to be significantly (*P*=0.0034) (~0.5 fold) down-regulated **H**) 2-OHE2 inhibited TNF-α-TNF-R1 expression and association in RA-FLS. Confocal imaging after immunostaining of TNF-α, *P*=0.0366 (Green) and TNF-R1, *P*=0.0282 (Red) in control and 2-OHE2 treated RA-FLS captured in 63 x oil-immersion objective. The nuclei were stained with DAPI (Blue). The fluorescence signal intensity was analyzed by ImageJ software and plotted as a bar Statistical analysis used the Student’s *t*-test, including Mann–Whitney *U*, and analysis of variance (ANOVA), values are presented as the mean ± SEM (*n* = 3). *P<0.05, **P<0.01, and ****P<0.0001. 1-OHE2, 2-hydroxyestradiol; Bax, Bcl2-associated X protein; Bcl2, B-cell lymphoma 2; Cytc, cytochrome complex; DAPI, 4',6-diamidino-2-phenylindole; MMP-9, matrix metalloproteinase-9; TNF-α, tumor necrosis factor alpha; TNF-R1, tumor necrosis factor receptor 1; qRT-PCR, quantitative real-time polymerase chain reaction; RA-FLS, rheumatoid arthritis fibroblast-like synoviocytes.

TNF-α persistently stimulates FLS, which results in alterations within the microenvironment, including the up-regulation of decoy receptors and molecules that inhibit apoptosis [47]. So, to investigate the effect of 2-OHE2 on apoptosis, western blot analysis of prominent proteins (Bax, Caspase3, Cyt C, Bcl2) and Caspase Glo 3/7 assay was performed. We found significant up-regulation of proapoptotic factors Bax (~2.3 fold) (Figure 4A), Caspase3 (~1.5 fold) (Figure 4B), and Cytc (~1.3 fold) (Figure 4C) after treatment with 2-OHE2 and simultaneously the decreased level of antiapoptotic factor Bcl2 (~0.42 fold) (Figure 4D). Further, up-regulation of Caspase 3/7 (~1.16 fold) indicated that 2-OHE2 induced the apoptotic stimulation in RA-FLS (Figure 4E). Subsequently, 2-OHE2 (1.25 μM) showed a significantly up-regulated mRNA expression of caspase3, Bax, and cytc (Figure 4F), suggesting the proapoptotic potential of 2-OHE2 in RA-FLS.

### 2-OHE2 inhibits the matrix degradation in RA-FLS

FLS expresses matrix metalloproteinases (MMPs) that degrade cartilage and bone collagen into immunodominant epitopes, maintaining the aggressive phenotype of the advancing pannus. Infiltrated immune cells overexpress, particularly MMP-9, induced by TNF-α, causing connective tissue degradation and triggering pathogenesis [48]. The level of MMP9 was therefore analyzed by WB after induction with 2-OHE2 (1.25 μM) and was found to be significantly decreased (~0.5 fold) in RA-FLS (Figure 4G).

### Reduced interaction of TNF-α with TNF-R1 in 2-OHE2 treated RA-FLS

TNF-α interacts with the cell surface receptor TNF-R1, a pivotal component in the inflammatory pathway. Upon binding, TNF-α activates multiple signal transduction pathways, thereby promoting IκBα degradation, NF-κB activation, and an increase in TNF-α levels. This intricate interaction is implicated in the pathogenesis of RA [3,49]. After treatment with 2-OHE2, both TNF-α and TNF-R1 expression and interaction decreased significantly in RA-FLS, as evidenced by immunofluorescence analysis. 2-OHE2 treatment exhibited significant inhibition of TNF-α expression evidenced by a reduction in green fluorescence (~0.5 fold) and TNF-R1 protein expression (~0.4 fold) indicated by a reduction in red fluorescence, compared with control RA-FLS. There was an appearance of strong expression (yellow color) in the merged image of the control cells formed by the interaction of red-colored TNF-R1 and green-colored TNF-α, which was less observed in the merged image of 2-OHE2 treated cells. (Figure 4H)

### CIA rat model establishment, amelioration of clinical severity, by 2-OHE2 treatment

**Figure 5:**
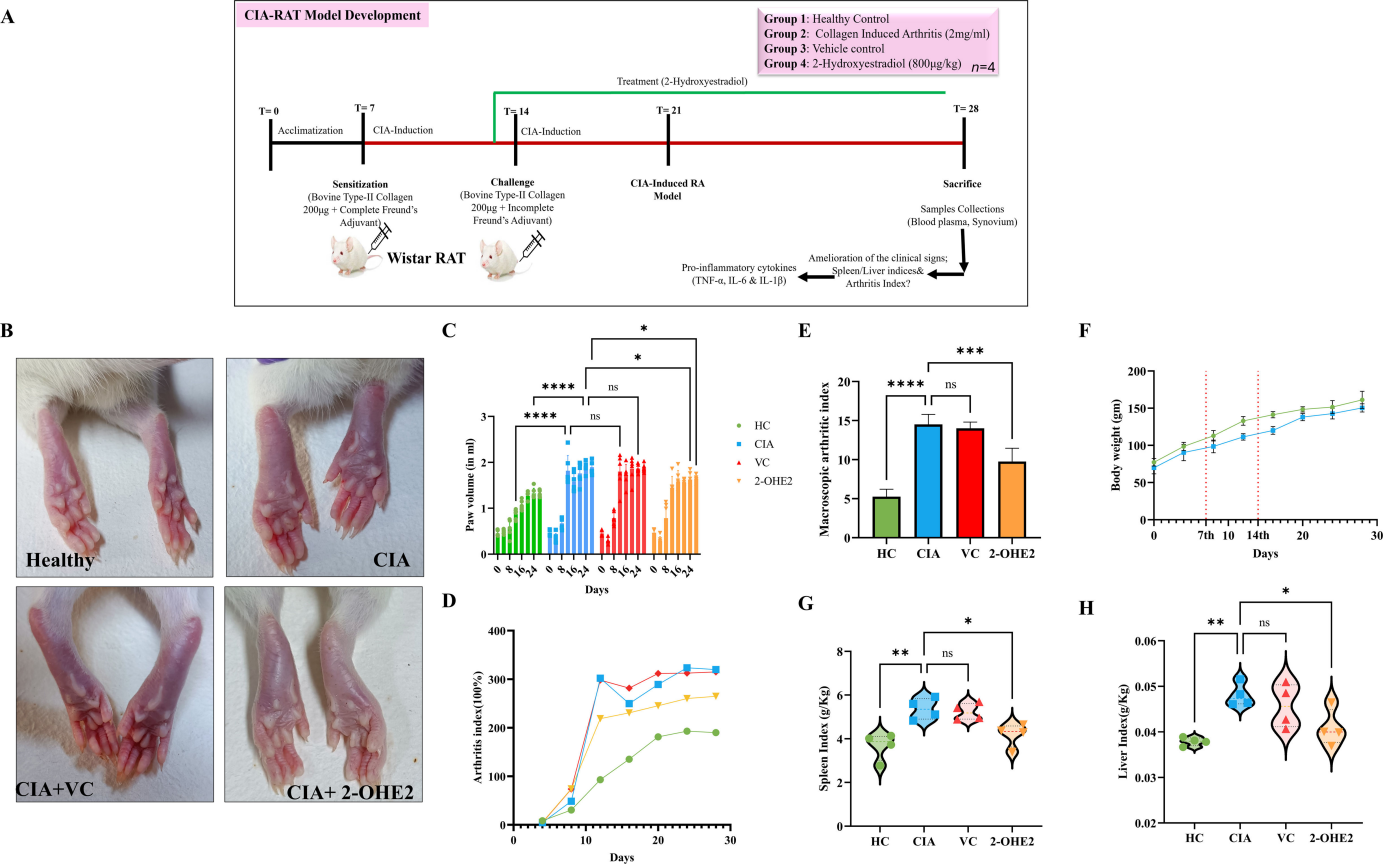
The effect of 2-OHE2 on collagen-induced arthritis rat model. **(A**) Graphical representation of CIA model development for the study. (**B**) Visual representative of hind paw image of the rats from each group, where edema and redness were reduced in Group 4 compared with Groups 2 and 3. (**C**) Relative representation of measured paw volume from day 0 to day 28, depicting the significant decrease in paw volume of Group 4 compared with Group 2. (**D**) The attenuation of the clinical Arthritis index (%) was reduced upon treatment of 2-OHE2 to the CIA group compared with control and indicates a significant difference between the groups. (**E**) The macroscopic arthritic score was measured on the 28th day, and a significant reduction in redness and swelling was found in Group 4 compared with Group 2. (**F**) Representation of the decreased weight in CIA compared with healthy showing the establishment of CIA. (**G**) The effect of 2-OHE2 on the spleen index of CIA rats at the time of sacrifice showed that the spleen weight relative to body weight (g/Kg) was significantly less in Group 4 compared with Group 2. Similarly, (**H**) liver index was also calculated and found less in Group 4. Statistical analysis used the Student’s *t*-test, including Mann–Whitney *U*, and analysis of variance (ANOVA). *P≤0.05, **P≤0.01, ***P≤0.001, and ****P<0.0001. 2-OHE2, 2-hydroxyestradiol; CIA, collagen-induced arthritis; Group 1, healthy or HC; Group 2, CIA; Group 3, CIA + VC; Group 4, CIA + 2-OHE2; HC, healthy control; VC, vehicle control.

The CIA rat model is widely used to mimic the RA condition (Figure 5A) [50]. We used this model to investigate the effects of 2-OHE2. Images of rat paws taken on the 28th day of all groups (Group 1 [HC], Group 2 [CIA], Group 3 [CIA + VC], and Group 4 [CIA + 2-OHE2]), before sacrification were shown (Figure 5B).

We observed that Group 4 showed less redness and swelling compared with Groups 2 and 3. The development of arthritis was quantified by measuring the paw volume with a plethysmometer twice weekly to confirm the onset of the disease. After day 16, paw volume decreased in Group 4, whereas paw volume increased in Group 2 (Figure 5C). CIA-induced arthritis was determined to progress successfully by measuring AI% between the groups. AI was more in Groups 2 and 3, which was decreased in Group 4 (Figure 5D). Similarly, the macroscopic arthritis score considerably decreased in Group 4 compared with Groups 3 and 2 (Figure 5E). Compared with the HC group, the CIA group had a significant increase in their AI and macroscopic scores. Additionally, the CIA group experienced a significant decrease in body weight, which indicated the establishment of the CIA model. (Figure 5F)

The proliferation of autoreactive B cells in RA is accompanied by increased immunoglobulin production and splenomegaly. In CIA rats, the liver and spleen are susceptible to chronic inflammation that can be measured as splenic and liver index [51]. In Group 2, the splenic index (Figure 5G) and liver index (Figure 5H) increased compared with normal rats, which has been found to be normalized by 2-OHE2, which exhibited protective effects.

### 2-OHE2 ameliorated inflammatory stress of synovium

**Figure 6:**
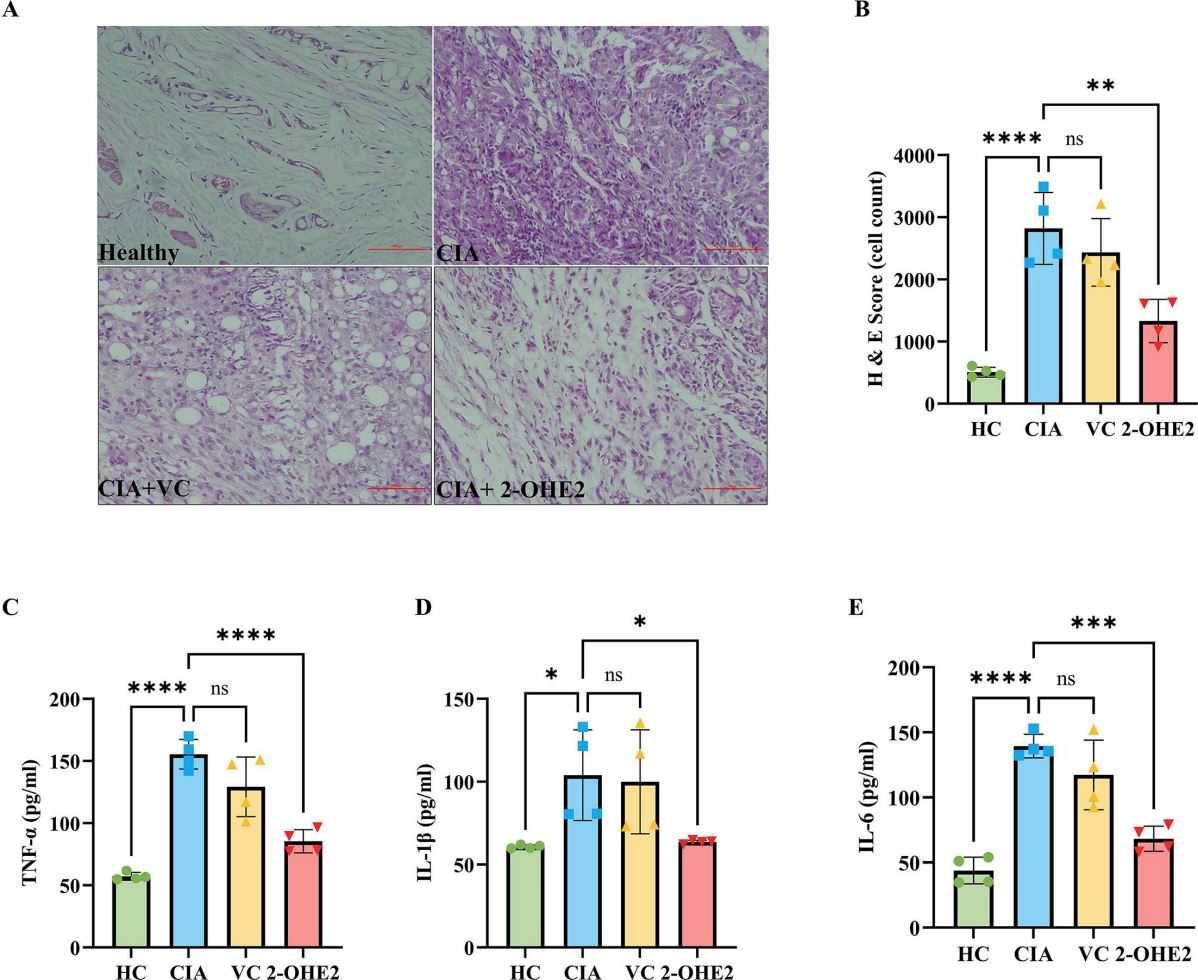
2-OHE2 reduces cellular infiltration and pro-inflammatory cytokines production in CIA rat. **(A**) The H&E staining shows decreased cell inflammation (purple color) in Group 4 compared with Groups 2 and 3. (**B**) The analysis of cell infiltration in the synovium was measured as cell count by Image J and represented as H&E score found to be down-regulated in Group 4 compared with Groups 2 and 3. The proinflammatory cytokine levels were measured by quantitative ELISA analysis in rat plasma in Groups 1 to 4, showing the down-regulation of **C**) TNF-α, (**D**) IL-1β, and (**E**) IL-6 levels in Group 4 compared with Groups 2 and 3. Statistical analysis used the Student’s *t*-test, including Mann–Whitney *U*, and analysis of variance (ANOVA). *P≤0.05, **P≤0.01, ***P≤0.001, and ****P<0.0001. 2-OHE2, 2-hydroxyestradiol; CIA, collagen-induced arthritis; ELISA, enzyme-linked immunosorbent assay; Group 1, healthy or HC; Group 2, CIA; Group 3, CIA + VC; Group 4, CIA + 2-OHE2; HC, healthy control; H&E, hematoxylin and eosin; IL-1β, interleukin 1 beta; IL-6, interleukin -6; TNF-α, tumor necrosis factor alpha; VC, vehicle control.

To further validate the anti-inflammatory activity of 2-OHE2, histological tests were performed on rat synovium by H&E staining (Figure 6A). The pink color represents cytoplasm, which correlates with the synovium’s inflammation. The purple color represents the number of nuclei present, used to determine the number of cells infiltrated into the given region and to quantify inflammation [50]. The H&E scan analysis revealed that the group injected with 2-OHE2 (Group 4) exhibited much less cell infiltration compared with Groups 3 and 2. (Figure 6B)

### Anti-inflammatory effect of 2-OHE2 by decreasing the levels of pro-inflammatory cytokines

Levels of pro-inflammatory cytokines were also measured by ELISA in the rat plasma of all groups. Down-regulation of pro-inflammatory cytokines (TNF-α, IL-1β, IL-6) was also revealed in rat plasma in Groups 4 compared with Groups 2 and 3 (Figure 6C, D and E). This supports the idea that Group 4 selectively regulated pro-inflammatory cytokines through the NF-kB cross-talk, significantly reducing inflammation in the CIA rats.

## Discussion

RA is an autoimmune inflammatory disease of joints, affecting females more than men. Sex hormones are related to immune response, with estrogens acting as enhancers [13]. Currently, the estrogen metabolism pathway is gaining interest since its various metabolites are reported to play a role in inflammatory diseases such as RA [52].

Metabolomics, as an emerging field in the arena of disease pathophysiology, is involved with the study of small molecule products of specific pathways known as metabolites [6]. The downstream metabolites of estrogen are termed CEs, formed due to the hydroxylation of estrogen [14]. Literature also suggests that estrogen metabolites get differentially regulated as the hydroxylation rate at specific carbon numbers gets altered in RA conditions [53]. Numerous studies reported the potency of estrogen, but negligible studies have explored the role of estrogen metabolites in inflammatory progression.

Genes regulating inflammation, cell survival, proliferation, and differentiation are primarily activated by TNF-α through activation of the NF-κB pathway. Canonical NF-κB activation leads to rapid expression of pro-inflammatory genes, whereas non-canonical pathways promote cell survival and proliferation in a sustained manner, on a slower timescale [42]. The report shows that TNF-α signaling is not effectively terminated in FLS, leading to an uncontrolled inflammatory response [49]. RA-FLS, described as ‘transformed’ cells, sharing morphologic features with tumor cells, such as resistance to apoptosis, increased rate of proliferation, metabolic shift, and high rate of inflammatory cytokines secretion [54,55]. Initiation of TNF-α signaling is prominent in RA-FLS to get these features that help in maintaining the disease environment.

It has been demonstrated that inhibition of TNF-α substantially lowers the level of inflammatory mediators and therefore, its inhibition can be formulated into therapeutic efficiency in RA [56]. Currently, small molecule inhibitors of TNF-α are becoming more popular than biologics due to their severe side effects [5], and 2-OHE2, as a metabolite of estrogen, is a self-molecule that has fewer chances of causing side effects when used as therapy [57]. The binding of TNF-α with TNFR triggers various intracellular signaling events that lead to the transcription of genes related to inflammation and apoptosis [48]. Reports show that certain metabolites can function as anti-inflammatory agents in diseases [16]. Earlier, in our in-silico studies, we screened estrogen metabolite 2-OHE2 that effectively targets TNF-α [8]. Therefore, we aimed to investigate the potential of the metabolite 2-OHE2 as an anti-inflammatory agent to target TNF-α in order to reduce RA pathogenesis. We validated our findings through biophysical studies: tyrosine quenching, thermal stability assay, and *in vitro* studies using RA-FLS followed by *in vivo* studies.

In the present study, the effectiveness of 2-OHE2, a small molecule inhibitor of TNF-α, has been revealed using a tyrosine quenching assay, leading to the conclusion that TNF-α was not readily available to interact with its receptor. Similarly, isothermal titration calorimetry (ITC) analysis [58] revealed *Kd* of ~12 μM with favorable thermodynamic parameters (Supplementary Figure S8)**,** suggesting an effective binding between 2-OHE2 and TNF-α. As a result, various aspects of TNF-α-initiated signaling cascades that are related to the pathogenesis of RA in RA-FLS were altered [54]. It was demonstrated that 2-OHE2 effectively inhibited inflammation by decreasing the level of p65 and p-p65 in both cytoplasmic and nuclear fractions and increasing the level of IκBα. Similarly, it initiated apoptosis by increasing the proapoptotic proteins (Bax, casp3, cytc) in RA-FLS and decreasing the antiapoptotic markers (Bcl2). This was further validated by the Caspase-Glo assay, which demonstrated the potential to induce apoptosis in RA-FLS. The expression of VEGF, a potent inducer of angiogenesis, and related adhesion and proliferation molecules were also found to be reduced by 2-OHE2 in RA-FLS. The scratch assay is used to measure basic cell migration parameters such as speed, persistence, and polarity, and to measure migratory capabilities of RA-FLS, which was found to be inhibited in the presence of 2-OHE2. Expression of the matrix degradation enzyme was also regulated by TNF-α signaling, which was found to be down-regulated at the protein level. The inclusion of 2-OHE2 during the interaction of TNF-α and TNF-R1 in RA-FLS was observed to provide resistance to their interaction, thus validated through immunofluorescence analysis, revealing less interaction of the two proteins in 2-OHE2-treated cells. We also performed a TNFR1 immunoprecipitation assay [59] and found a low expression of RIP1 after induction of 2-OHE2, suggesting a low activation of TNF-α signaling (Supplementary Figure S9).

In addition, the therapeutic potential of 2-OHE2 was confirmed in the CIA rat model. Results indicate that Group 4, which was treated with 2-OHE2, experienced a protective effect in CIA rats. This was evidenced by a reduction in paw volume and macroscopic arthritic score. We also analyzed the levels of cytokines, particularly TNF-α, IL-6, and IL-1β, in the plasma of rats from different groups. Our analysis suggests that 2-OHE2 has down-regulated pro-inflammatory cytokines, indicating its efficacy as an anti-inflammatory small molecule. These findings provide additional evidence of the therapeutic potential of 2-OHE2 that may mitigate inflammation in a CIA rat model.

Therefore, this metabolite targets TNF-α-initiated signaling cascades by inhibiting inflammation, promoting apoptosis, and reducing key proteins associated with RA pathogenesis in RA-FLS. Our findings thus demonstrated that the metabolite 2-OHE2 may have the potential to be a safe and effective therapeutic candidate for treating RA due to its endogenic nature, and it might offer a novel approach for developing targeted therapies against RA progression with fewer side effects.

## Conclusion

Inhibition of protein–protein interaction (PPI) by small molecules is vital as they target the binding site’s ‘hot spots’, providing the majority of the binding energy, and small biomolecules explored to inhibit TNF-α, resulting in the most efficient strategies to decrease inflammation. Identifying 2-OHE2 as a novel estrogen metabolite was critical in reducing inflammation via targeting TNF-α, which might effectively inhibit TNF-α signaling and ameliorate disease symptoms. Thus, the study evaluated the potential of identified metabolites in monitoring cellular inflammatory mechanisms in RA pathobiology.

**Clinical Perspective**

- **Background as to why the study was undertaken:** The development of rheumatoid arthritis (RA) involves various proinflammatory cytokines, with TNF-α being the most prominent. Our previous in-silico study (2022) identified 2-OHE2, an estrogen metabolite, as a promising candidate that effectively targets TNF-α. Therefore, we aimed to investigate the potential of the metabolite 2-OHE2 as an anti-inflammatory agent to target TNF-α in order to reduce RA pathogenesis.
- **A brief summary of the results:** The effectiveness of 2-OHE2, a small molecule inhibitor of TNF-α, has been demonstrated using a tyrosine quenching assay, concluding that TNF-α was not readily available to interact with its receptor. It was shown that 2-OHE2 effectively reduced inflammation by decreasing NF-κB and initiating apoptosis. The therapeutic potential of 2-OHE2 was also confirmed in the CIA rat model.
- **The potential significance of the results to human health and disease:** Our findings thus demonstrated that the metabolite 2-OHE2 may have the potential to be a safe and effective therapeutic candidate for treating RA due to its endogenic nature, and it might offer a novel approach for developing targeted therapies against RA progression with fewer side effects.

## Supplementary material

Online supplementary material 1

## Data Availability

For all original data and protocol, please contact Dr Sagarika Biswas (Sagarika.biswas@igib.res.in).

## Open Access

This article has been published open access under our Subscribe to Open programme, made possible through the support of our subscribing institutions, learn more here: https://portlandpress.com/pages/open_access_options_and_prices#conditional

## Abbreviations

ACCPA: anti-citrullinated protein/peptide antibody
BSA: bovine serum albumin
Bax: Bcl2-associated X protein
Bcl2: B-cell lymphoma 2
Cytc: cytochrome complex
DMSO: dimethyl sulfoxide
EDTA: ethylenediamine tetraacetic acid
FPLC: fast protein liquid chromatography
GAPDH: glyceraldehyde-3-phosphate dehydrogenase
IPTG: isopropyl β-d-1-thiogalactopyranoside
IkBα: nuclear factor of kappa light polypeptide gene enhancer in B-cells inhibitor α
KH2PO4: potassium dihydrogen phosphate
MMP-9: matrix metalloproteinase-9
NF-κB: nuclear factor kappa-light-chain-enhancer of activated B cells
NaCl: sodium chloride
Na2HPO4: sodium hydrogen phosphate
Ni2SO4: nickel(II) sulfate
OD: optical density
PBS: phosphate-buffered saline
PCR: polymerase chain reaction
RF: rheumatoid factor
SDS-PAGE: sodium dodecyl-sulfate polyacrylamide gel electrophoresis
TCEP: tris(2-carboxyethyl)phosphine
TNFR1: tumor necrosis factor receptor 1
VEGF: vascular endothelial growth factor
