# Supplementary material for: Evaluating the role of 2-hydroxyestradiol in modulating TNF-α signaling and its implications in rheumatoid arthritis
Source: Clin Sci (Lond). 2025 Sep 22;139(18):979–95. doi: 10.1042/CS20241917 (PMC12599235; doi:10.1042/CS20241917)
Supplement: Online supplementary material 1 [file cs-139-18-CS20241917-s001.docx]

**Supplementary File**

**Evaluating the Role of 2-Hydroxyestradiol in Modulating TNFα Signaling and Its Implications in Rheumatoid Arthritis**

**Prachi Agnihotri^1,2^, Mohd Saquib^1,2^, Ajit Kumar^1,2^, Lovely Joshi^1,2^, Debolina Chakraborty^1,2^, Ashish Sarkar^1,2^, Vijay Kumar^3^, Sagarika Biswas^1,2^***

^1^Council of Scientific & Industrial Research (CSIR)-Institute of Genomics and Integrative Biology, Mall Road, Delhi University Campus, Delhi, India,110007

^2^Academy of Scientific and Innovative Research (AcSIR), Ghaziabad-201002, India

^3^All India Institute of Medical Sciences, Ansari Nagar, New Delhi - 110029, India

# *Corresponding author

Dr. Sagarika Biswas, Ph.D.

Chief Scientist

Integrative and Functional Biology Department CSIR- Institute of Genomics & Integrative Biology, Mall Road, Delhi-110 007, India

Tel: +91 11 27667602

& Fax #: +91-11-27667471, #9818004740

E-mail: [sagarika.biswas@igib.res.in](mailto:sagarika.biswas@igib.res.in) (ORCID ID: 0000 0002 2017 1670)

**Supplementary materials and methods:**

**Isothermal Titration Calorimetry (ITC)**

ITC were conducted at 25°C using a Microcal PEAQ-ITC apparatus by MicroCal, LLC. Both the protein and 2-hydroxyestradiol (2-OHE2), were prepared in the same binding buffer with consistent DMSO levels (0.05%). The 2-OHE2, dissolved in 100% DMSO. 2-OHE2, at the concentration of ~500 µM, titrated into 300 µL of ~20 μM TNFα across 19 injections of 2 µL each. The control heat was automatically subtracted from the titration using the fitted offset feature, and the integrated heat data from the titration were analyzed using the MicroCal PEAQ-ITC analysis software. A one set site binding model was employed to fit the isotherms, and thermodynamic parameters were derived.

**Western blotting of Nucleo-Cytoplasmic extraction of RA-FLS**

RA-FLS were treated with 2-hydroxyestradiol (2-OHE2) (1.25 μM) for 24 h at 37 °C. The cells were harvested, and the proteins of cytoplasm and nucleus were extracted respectively by nuclear and cytoplasmic protein extraction kit (NE-PER Nuclear and cytoplasmic extraction reagent, Thermo-78833); 20 ug of proteins were separated on SDS-PAGE as described earlier. The membranes were then incubated with primary antibodies that recognized p65 (Santa Cruz, USA, 1:1000), and p-p65 (CST, USA, 1:1000). GAPDH and histone were used as loading controls.

**TNFR1 immunoprecipitation and signaling assay:**

RA-FLS were incubated with 2-hydroxyestradiol (2-OHE2) (1.25 μM) for 24 h at 37 °C. Cells were then lysed for 1 h in cold lysis RIPA buffer, and cell supernatant was harvested. TNFR1 immunoprecipitation was performed by incubating supernatant with 5 μg anti-TNFR1 antibody overnight while rotating at 4 °C in the IP column (Catch and Release® v2.0 Reversible Immunoprecipitation System). The column was subsequently washed 3× in wash buffer, and then the elution was taken. Flow-through, wash, and elution were then loaded on an SDS-PAGE. Gels were then transferred to NC membrane and blocked in 5% BSA for 1 h. RIP-1 (santa cruz) and TNFR1 (CST) were then detected by Western blotting using anti-mouse-and anti-rabbit secondary antibodies.

**Results:**

**Downregulated expression of p65 and p-p65 in nucleo-cytoplasmic extracts in** **2-OHE2-treated RA-FLS**

The activation of the NF-κB pathway is regulated by the phosphorylation of p65, which leads to its translocation into the nucleus. This process results in the activation of inflammation and other cellular signaling pathways. Therefore, in order to examine the influences of 2-OHE2 on NF-κB activity, we extracted proteins from the cytoplasm and nucleus, respectively, after RA-FLS were treated with 2-OHE2 and detected the expression of p65 and p-p65 in the cytoplasm and nucleus, respectively, and found decreased expression (**Figure S4)**. Immunocytochemistry confirmed the analysis of p65 protein expression after 2-OHE2 induction in RA-FLS. Treated cells showed significantly lower p65 levels, indicated by reduced green fluorescence compared to untreated control cells. (**Figure S5)** This observation suggests that 2-OHE2 leads to decreased activation of NF-κB pathway.

**Thermodynamic Analysis of 2-hydroxyestradiol TNFα Interaction**

We utilized ITC to offer precise quantitative insights into the molecular interaction between 2-OHE2 and TNFα. This technique not only accurately determines the affinity constant but also reveals comprehensive thermodynamic parameters and binding stoichiometry, crucial for understanding the underlying mechanisms. The heat released during the titration of the ligand into the TNFα solution showed excellent agreement with ideal binding curve, suggesting the presence of a singular binding site type. The measured dissociation constants (Kd) fell within the range of 12±2.55μM for 2-OHE2 (**Figure S8).** The interaction between 2-OHE2 and TNFα was driven by both favorable enthalpic and entropic contributions. The ΔG for the interaction was mainly negative due to compensation between enthalpy and entropy.

**Proximal signaling of TNFα inhibited by 2-OHE2 in RA-FLS through the downregulation of RIP1 expression**

To evaluate the impact of the 2-OHE2 on the TNFR1 signaling pathway, we initially focused on the early events related to TNFR1 by measuring the recruitment of signaling proteins, specifically RIP-1, which is identified by its ubiquitination. In control RA-FLS cells, we noted an increase in RIP-1 recruitment to TNFR1, as demonstrated by activated TNFα signaling. However, pretreatment of the cells with 2-OHE2, the recruitment of RIP-1 to the TNFR1 signaling complex was found to be downregulated. This suggests that this metabolite interfered with the TNFα stimulated TNFR1's ability to recruit downstream proteins. **(Figure S9)**

**Supplementary Figure & Table legends:**

**Figure S1:** Fast protein liquid chromatography (FPLC) trace of eluted homotrimer of TNFα. TNF-α, Tumor necrosis factor alpha

**Figure S2:** Western Blot analysis of eluted fractions of TNFα. TNF-α, Tumor necrosis factor alpha

**Figure S3:** The SDS-PAGE analysis of Fast protein liquid chromatography (FPLC) purified TNFα protein. SDS-PAGE, Sodium dodecyl sulfate-polyacrylamide gel electrophoresis; TNF-α, Tumor necrosis factor alpha.

**Figure S4:** Effect of 2-OHE2 on nuclear factor κB (NF-κB) activity in RA-FLS. Cells were treated with 1.25 μM 2-OHE2 for 24 h **(A)** Proteins of the cytoplasm were extracted and NF-κBp65 (p=0.0082) and GAPDH was detected by Western blotting. **(B)** Proteins of the nucleus were extracted and p-p65 (p=0.0080) and histone was detected by Western blotting. 2-OHE2, 2-hydroxyestradiol; RA-FLS, Rheumatoid Arthritis fibroblast-like Synoviocytes; Statistical analysis used the Student’s t-test, including Mann–Whitney U , values are presented as the mean ± SEM (n = 3). ** = < 0.01, *** = < 0.001 and **** = < 0.0001

**Figure S5:** Confocal imaging after immunostaining of p65, downregulated expression was found (p=0.0041) (Green) in 2-OHE2-treated compared to control RA-FLS, captured in a 63x oil-immersion objective. The nuclei were stained with DAPI (Blue). The Fluorescence signal intensity was analyzed by ImageJ software and plotted as a bar graph. 2-OHE2, 2-hydroxyestradiol; RA-FLS, Rheumatoid Arthritis fibroblast-like Synoviocytes; DAPI, 4',6-diamidino-2-phenylindole; Statistical analysis used the Student’s t-test, including Mann–Whitney U, values are presented as the mean ± SEM (n = 3). * = p < 0.05, ** = < 0.01, and **** = < 0.0001.

**Figure S6: A)** The expression level of Ki67 observed in RA-FLS by Western Blot, pre-treated with 1.25 μM 2-OHE2 for 24 h, significantly (p=0.0002) downregulated (~0.6 fold) **B)** The expression level of N-CAD observed in RA-FLS by Western Blot, pre-treated with 1.25 μM 2-OHE2 for 24 h, significantly (p=0.0002) downregulated (~0.5 fold). 2-OHE2, 2-hydroxyestradiol; RA-FLS, Rheumatoid Arthritis fibroblast-like Synoviocytes; Ki67, Marker of Proliferation Kiel 67; N-CAD, Neural Cadherin; Statistical analysis used the Student’s t-test, including Mann–Whitney U,  values are presented as the mean ± SEM (n = 3). ** = < 0.01, *** = < 0.001 and **** = < 0.0001

**Figure S7: A)** The expression level of FGF observed in RA-FLS by Western Blot, pre-treated with 1.25 μM 2-OHE2 for 24 h, significantly (p=0.0394) downregulated (~0.6 fold) **B)** The expression level of CDH11 observed in RA-FLS by Western Blot, pre-treated with 1.25 μM 2-OHE2 for 24 h, significantly (p=0.0436) downregulated (~0.8 fold). **C)** The expression level of VCAM-1 observed in RA-FLS by Western Blot, pre-treated with 1.25 μM 2-OHE2 for 24 h, significantly (p=0.0194) downregulated (~0.75 fold). 2-OHE2, 2-hydroxyestradiol; RA-FLS, Rheumatoid Arthritis fibroblast-like Synoviocytes; FGF, Fibroblast Growth Factor; CDH11, Cadherin-11; VCAM-1, Vascular Cell Adhesion Molecule 1; Statistical analysis used the Student’s t-test, including Mann–Whitney U, values are presented as the mean ± SEM (n = 3). ** = < 0.01, *** = < 0.001 and **** = < 0.0001

**Figure S8:** Representative ITC of 2-OHE2 and TNF-α interaction: The experimental findings revealed a dissociation constant of approximately 12 μM for TNF-α binding to 2-hydroxyestradiol. The heat of interaction was obtained after the raw data were normalized and integrated using the Microcal PEAQ-ITC program. The heat of dilution was subtracted from the raw data using the fitted offset option. The red line represents the best fit obtained by a nonlinear least-squares data fit to two independent sites binding model for the TNF-α construct. The data were fitted using one set of site models. 2-OHE2, 2-hydroxyestradiol; ITC, Isothermal titration calorimetry; TNF-α, Tumor necrosis factor alpha.

**Figure S9:** Western blots measuring RIP1 following TNFR1 immunoprecipitation from RA-FLS cells treated with 2-OHE2. RIP1 was found to be downregulated along with TNFR1 in the elution of cell lysates treated with 2-OHE2. 2-OHE2, 2-hydroxyestradiol; RA-FLS, Rheumatoid Arthritis fibroblast-like Synoviocytes; RIP1, Receptor-interacting serine/threonine protein kinase 1; TNF-R1, Tumor necrosis factor receptor 1.

**Supplementary Table 1:** The clinical demography characteristics of RA patients.

**Supplementary Table 2.** The amplification primers sequences of human-specific genes.

**Figure S1:**


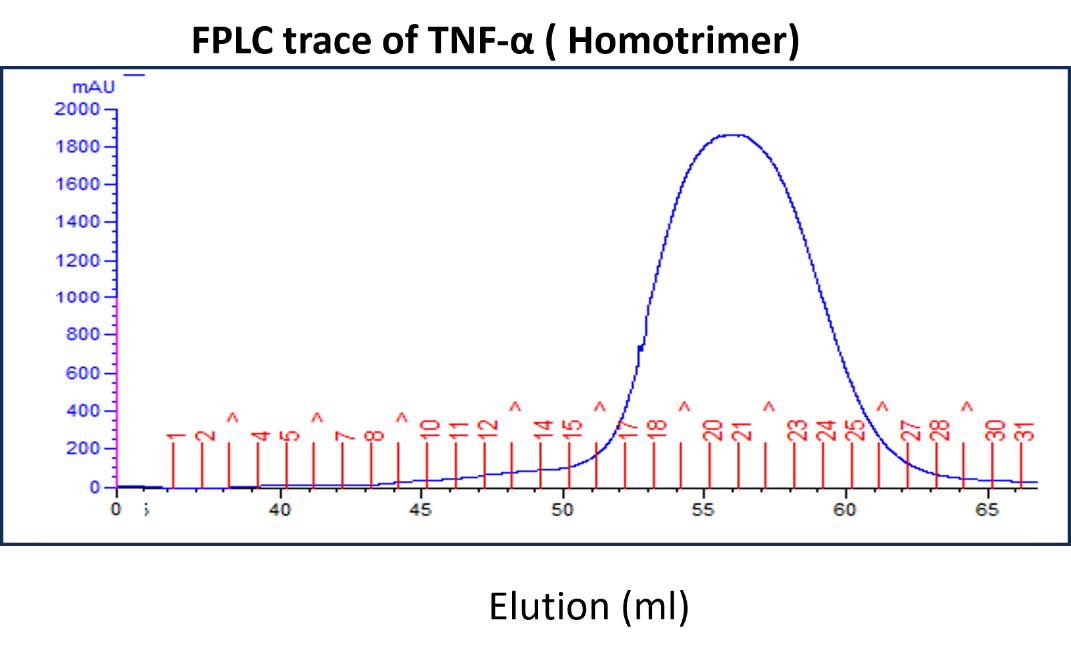


**Figure S2:**


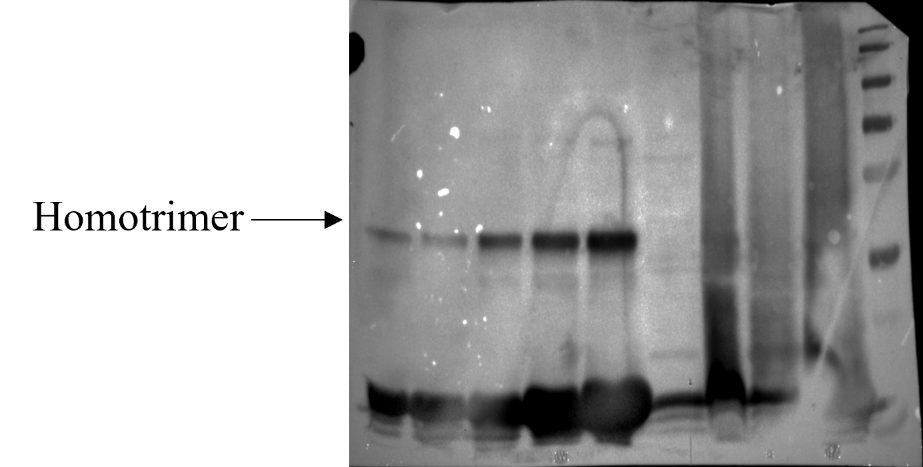


**Figure S3:**


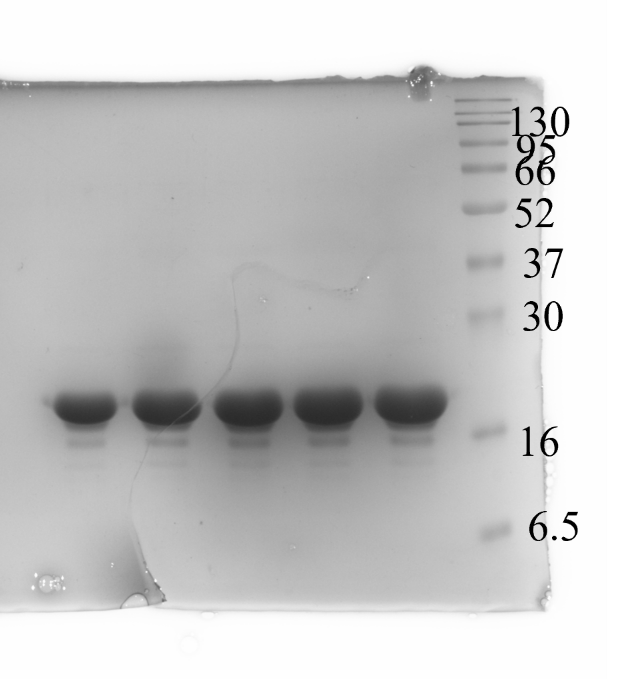


**Figure S4:**


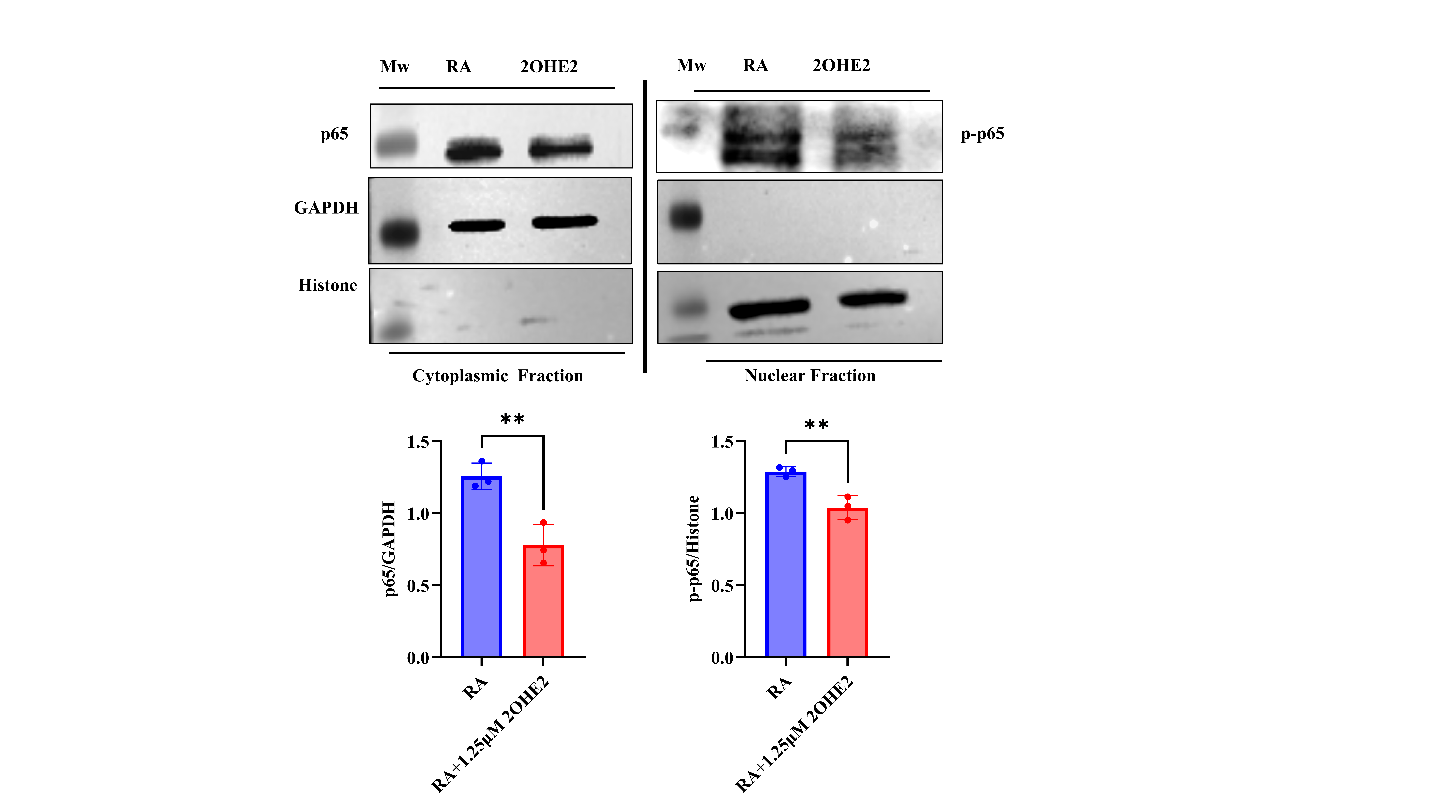


**Figure S5**


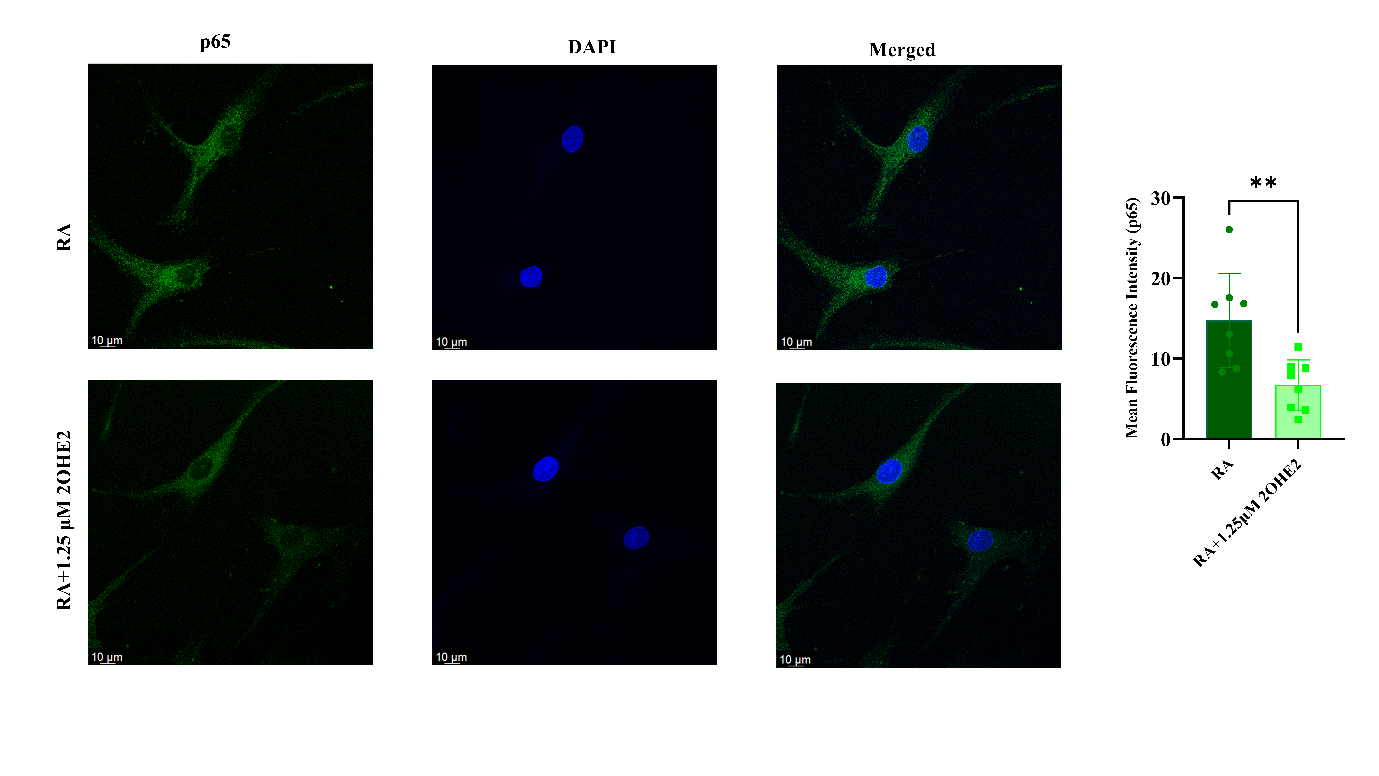


**Figure S6:**


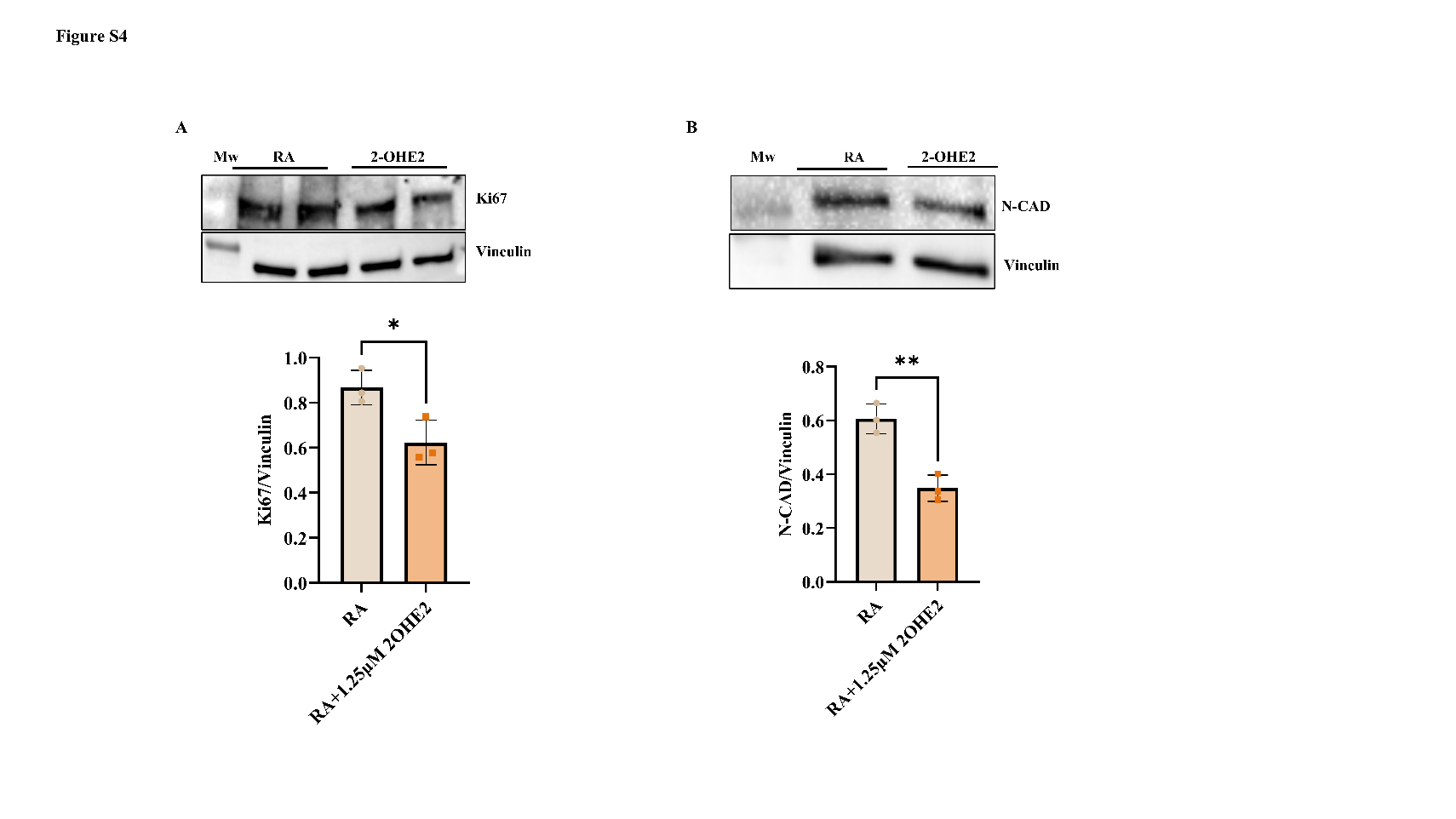


**Figure S7:**


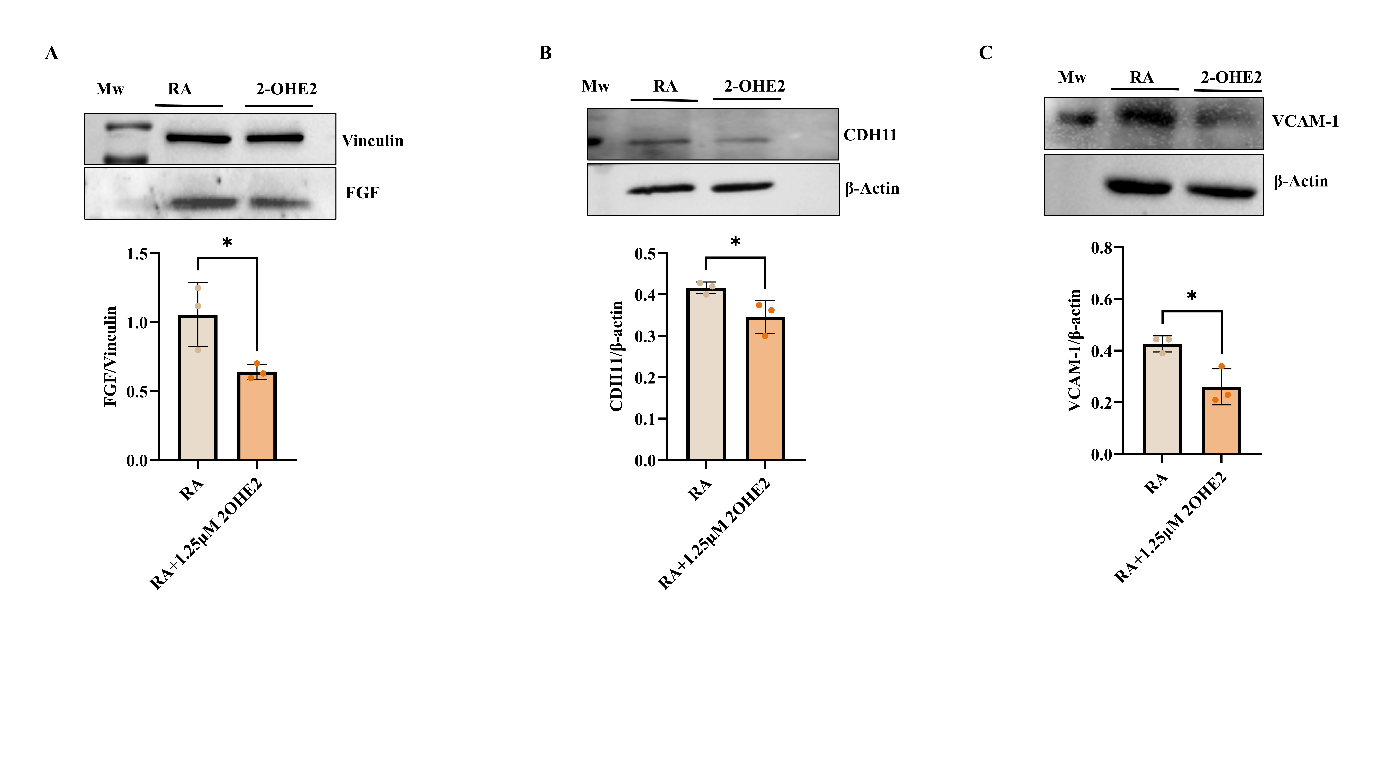


**Figure S8:**


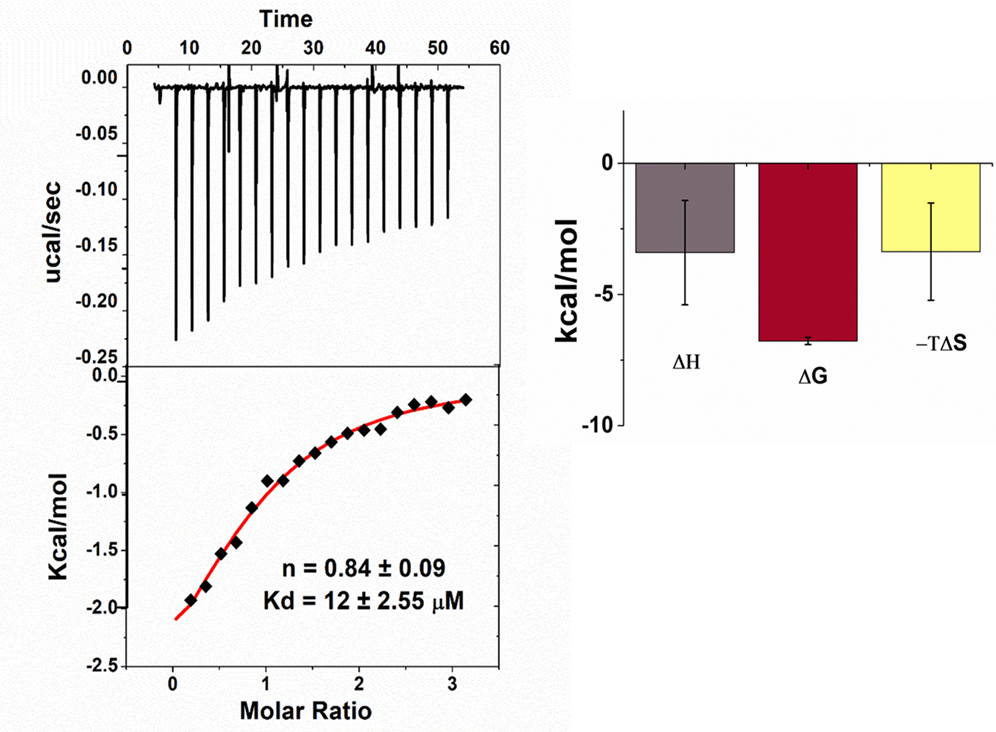


**Figure S9:**


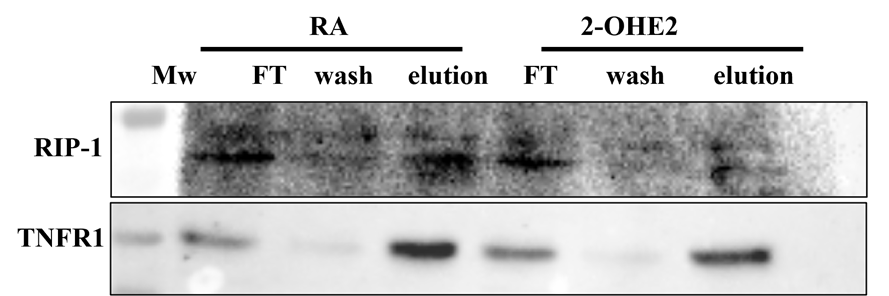


**Supplementary Table 1:**

| **S. No.** | **Patient’s characteristics** | **RA (n=8)** |
| --- | --- | --- |
| 1 | Age (yrs.) | 50 ± 5 |
| 2 | Sex (Female, Male) | F (5), M (3) |
| 3 | ESR (mm/hr) | 35 ± 5 |
| 4 | RF (+ve/-ve) | +ve |
| 5 | CRP (mg/L) | 80 ±15 |
| 6 | Tender Joint | 20±6 |
| 7 | Swollen joints | 10±4 |
| 8 | DAS-28 score | 6 ± 0.5 |
| 9 | Disease duration yrs. | 10 ± 5 |
| 10 | Medication (Yes/No) | Yes |

*RA: Rheumatoid arthritis; ESR: Erythrocyte sedimentation rate; RF: Rheumatoid factor; CRP: C-reactive protein; DAS-28: Disease Activity Score-28. The values are expressed as Mean ±SD

**Supplementary Table 2.**

| **Sr.** | **Gene Name** | **Primer Sequence Forward** | **Primer Sequence Reverse** |
| --- | --- | --- | --- |
| 1 | Full length TNFα | 5`ACACTCGGATCCGTGCGTAGCAGCAGCC 3` | 5`ACACTCCTCGAGTTACAGCGCAATAATACCG 3` |
| 2. | Caspase3 | 5`CATGGAAGCGAATCAATGGACT 3` | 5`CTGTACCAGACCGAGATGTCA 3` |
| 3 | Cyt c | 5`TTTGGTTGCACTTACACCGG 3` | 5`GGACGTCCCCACTCTCTAAG 3` |
| 4 | Bax | 5`CCCGAGAGGTCTTTTTCCGAG 3` | 5`CCAGCCCATGATGGTTCTGAT 3` |
| 5 | VEGF | 5`TGCAGATTATGCGGATCAAACC3` | 5` TGCATTCACATTTGTTGTGCTGTAG 3’ |
| 6 | VCAM | 5` GTGTCATGGGCTGTGAATCC 3` | 5` CAAAACTCACAGGGCTCAGG 3` |
| 7 | CDH11 | 5`CCACAATCGGCATCAGGAAG 3` | 5` GCCGTGTCATCCTTGTCATC 3` |
| 8 | FGF | 5`AATGTGTTACGGATGAGTGTTTC3` | 5`CTTTCTGCCCAGGTCCTGTT3` |
| 9 | IL-6 | 5’GGTACATCCTCGACGGCATCT3` | 5`GTGCCTCTTTGCTGCTTTCAC-3` |
| 10 | TNF-α | 5`CCCCAGGGACCTCTCTCTAATC3` | 5`GGTTTGCTACAACATGGGCTACA 3` |
| 11 | IL-1β | 5′AAACAGATGAAGTGCTCCTTCCAGG 3′ | 5′ GGAGAACACCACTTGTTGCTCCA 3′ |
| 13 | GAPDH | 5` GAAGGTGAAGGTCGGAGTC 3` | 5` GAAGATGGTGATGGGATTTC 3` |
